# An Unexpected Controlled New Oxidant: SO_4_^.–^

**DOI:** 10.1038/srep20163

**Published:** 2016-02-01

**Authors:** Cui-Bing Bai, Nai-Xing Wang, Xing-Wang Lan, Yan-Jing Wang, Yalan Xing, Jia-Long Wen, Xue-Wang Gao, Wei Zhang

**Affiliations:** 1 Technical Institute of Physics and Chemistry, Chinese Academy of Sciences, Beijing, 100190, China; 2 Department of Chemistry, William Paterson University of New Jersey, 300 Pompton Road, Wayne, New Jersey 07470, United States

## Abstract

A controlled new oxidant sulfate radical anion (SO_4_^**.–**^) was found and it can be easily prepared by mixing Na_2_S_2_O_4_ and TBHP with stirring. In this new metal-free oxidation system (Na_2_S_2_O_4_/TBHP), SO_4_^**.–**^ can be used as a controllable oxidant to oxidize various aromatic alcohols to the corresponding aldehydes in good yields without any acid formation at room temperature. SO_4_^**.–**^ was determined by a DMPO (5,5-dimethyl-1-pyrroline-*N-*oxide) spin-trapping EPR method at room temperature on a Bruker E500 spectrometer and the results suggested that SO_4_^**.–**^ was generated in this transformation.

The selective oxidation of alcohols to the corresponding aldehydes is highly relevant reactions in organic chemistry^1^,^2^, and is a fundamental and pivotal transformation for laboratory research and industrial manufacturing^3^. To date, great efforts have been continuously devoted to the development of excellent oxidation systems for alcohol oxidations^4^,^5^,^6^. Classically, oxidations of alcohols are performed with inorganic oxidants (notably chromium and manganese oxides), Swern reagents and Dess-Martin reagents (Fig. 1)^7^,^8^. However, these stoichiometric oxidants suffer from many drawbacks such as relatively expensive reagents and difficulties in handling heavy-metal wastes^9^. From the viewpoints of environmental concern, more and more attention has been paid towards the development of catalytic aerobic alcohol oxidation methodologies^10^,^11^. In the past few decades, transition metal-catalyzed oxidation of alcohols to aldehydes or ketones has been established^12^,^13^. For example, the catalytic systems employing different transition metals in combination with TEMPO (2,2,6,6-tetramethyl-piperidyl-1-oxy) or TBHP (*tert*-butyl hydroperoxide)^14^,^15^. Although many of these methods were effective for the preparation of the desired products, additional base, transition metals and high reaction temperatures are often needed as shown in Fig. 1^9^.

Recently, a transition-metal-free catalytic system for oxidations catalyzed by TEMPO/NaNO_2_ has attracted special attention due to their outstanding catalytic efficiency^16^,^17^. However, these methods have some drawbacks, such as the use of O_2_ often requires work in autoclaves^18^. And high reaction temperatures are often needed. Despite lots of precedents and advancements in alcohol oxidation strategies recently, there have been few reports on the metal-free oxidation system for oxidation catalyzed by TBHP under mild conditions. Thus, the search for developing highly efficient oxidation system without metals for the oxidation of alcohols remains an area of intensive interest.

As we all know, Na_2_S_2_O_4_ (sodium dithionite) is one of the most widely used strong reducing agents in organic chemistry. For example, in the synthesis of the chiral NADH models, Na_2_S_2_O_4_ is often used as the most effective reducing agent^19^,^20^,^21^. Na_2_S_2_O_4_ is an effective reagent for the reduction of aldehydes and ketones to the corresponding alcohols^22^. As a continuation of our research on TBHP and Na_2_S_2_O_4_^19^,^20^,^21^,^23^, in a accidental opportunity, we found that TBHP/Na_2_S_2_O_4_ can oxidize aromatic alcohols to corresponding aldehydes without any acid formation.

Furthermore, sulfate radical anion (SO_4_^.–^) can be formed by thermal activation, UV photolysis and transition metal catalysis^24^,^25^,^26^,^27^,^28^. In this paper, a controlled new oxidant sulfate radical anion (SO_4_^**.–**^) was found and it can be easily prepared by mixing Na_2_S_2_O_4_ and TBHP with stirring. To the best of our knowledge, this is first report that sulfate radical anion can be used as controllable oxidant to oxidize various aromatic alcohols to the corresponding aldehydes in good yields without any acid formation.

## Results and Discussion

We initially investigated the reaction between 4-methoxybenzyl alcohol (**1a**) with Na_2_S_2_O_4_ in the presence of 1 equiv of TBHP in EtOAc at room temperature for 12 h (Table 1, entry 1). To our delight, the Na_2_S_2_O_4_/TBHP oxidation system showed good catalytic activity for oxidation of **1a** to *p*-anisaldehyde (**2a**) with a good yield of 60% at room temperature (entry 1). Having a promising result in hand, we then optimized the reaction conditions. The initial study was carried out using 4-methoxybenzyl alcohol (**1a**) as the model substrate. The results were summarized in Table 1. It was found that the reactivity of the oxidation reaction was the best when molar ratio of Na_2_S_2_O_4_/TBHP/substrate was 2:4:1 (entry 4), while decreasing of the ratio led to lower reactivity (entry 1–3). Increasing the amount of aqueous TBHP or using anhydrous TBHP did not improve the yield of compound **2a**, only 81% and 78%, respectively (entries 4 and 5). Further blank experiments confirmed that the substrates were unreactive in the absence of either TBHP or Na_2_S_2_O_4_ (entries 6 and 7). The fact indicated that both TBHP and Na_2_S_2_O_4_ were essential for the oxidation. When other different oxidizing agents were used, such as NBS, BPO and DTBP, the yields were not high enough compared with the usage of TBHP (entries 9–11). In addition, no oxidation product was produced when H_2_O_2_ was used under this condition (entry 8). A profound solvent effect on the reactions was observed (entries 12–16). When the reaction was carried out in various solvents such as CH_3_CN, CH_2_Cl_2_, CHCl_3_, THF, cyclohexane and EtOAc, the best yield was observed using EtOAc. Hence, EtOAc was considered as an optimal solvent.

**Figure 2:**
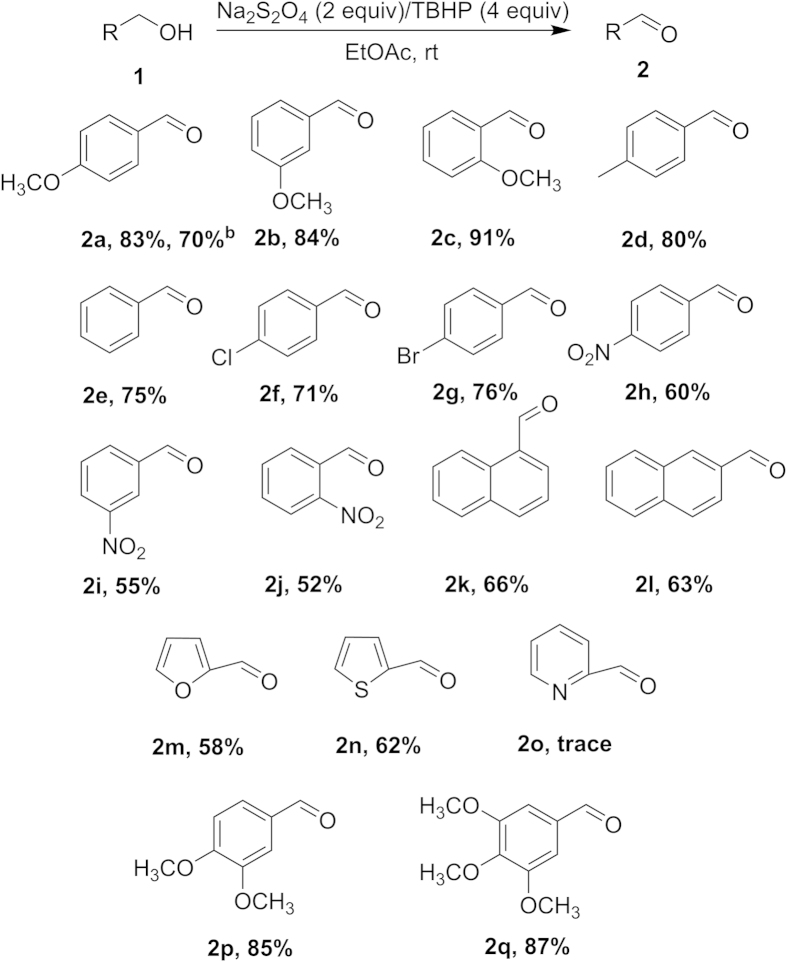
Oxidation of substituted benzylic and other aromatic alcohols.

To probe the efficiency of the Na_2_S_2_O_4_/TBHP oxidation system, the oxidation of various aromatic alcohols to the corresponding aldehydes was studied under the optimized conditions as summarized in Fig. 2. By the comparison of spectral data (^1^H and ^13^C NMR) with the authentic samples, all the aldehydes were well characterized. Benzylic alcohols with electron-donating substituents (e.g., methoxy) can be oxidized into corresponding aldehydes in excellent yields. However, in case of nitro-substituted benzylic alcohols, the yield was comparatively less than that of the electron-donating counterparts (**2h**). Under the same conditions, for aromatic alcohols with methoxy-substituted (**2a**–**2c**) and nitro-substituted (**2h**–**2j**) benzene ring, *para-*, *ortho-* and *met*a- aromatic alcohols could be converted into the corresponding products with good yields, respectively. Aromatic alcohols substituted with halogen gave satisfactory yields under the optimized conditions (**2f** and **2g**). Some other aromatic substrates such as furfurol and 2-thiophene methanol were readily oxidized to furfural and 2-thiophenecarboxaldehyde, respectively (**2m**, **2n**). However, only trace oxidation product was obtained with pyridin-2-ylmethanol (**2o**). In addition, we conducted a large-scale (10 mmol) reaction, and the results showed that this new oxidation system was also very effective even on the gram scale (**2a**^b^). In fact, we have previously studied some substrates with 2° aromatic alcohols. We found that the desired product acetophenone can be obtained in 53% yield. Unfortunately, this system is not good for controllable oxidation of fatty alcohols to their corresponding aldehydes.

**Figure 3:**
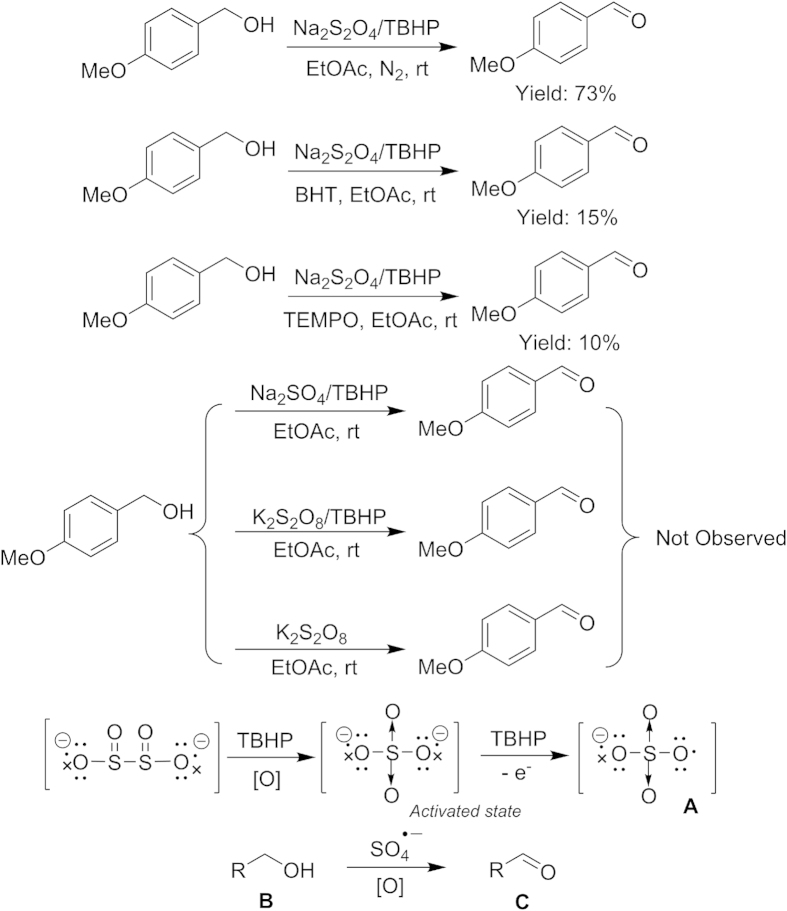
Plausible reaction mechanism.

The reaction was performed in the presence of Na_2_S_2_O_4_/TBHP under N_2_ atmosphere (Fig. 3) and the results showed that TBHP as the terminal oxidant in this reaction. Furthermore, the reaction yield dropped when radical inhibitor BHT (butylated hydroxytoluene) and TEMPO (2,2,6,6-tetramethylpiperidin-1-yloxyl) was added, respectively (Fig. 3). It indicated that the reaction should undergo a radical process. EPR spin trapping using the commonly used spin trap, 5,5-dimethyl-1-pyrroline-*N-*oxide (DMPO), has been employed in the detection of SO_4_^**.–**^ (see Supplementary Figs S1 and S2 online). A quartet of signals were detected during the reaction process, and the results suggested that SO_4_^**.–**^ was generated in this transformation^29^. As shown in Fig. S1 and Fig. S2, with the prolonged reaction time, the concentration of SO_4_^**.–**^ was also gradually increasing in ethyl acetate or acetonitrile.

In fact, we have used sodium sulfate (Na_2_SO_4_) in place of dithionate (Na_2_S_2_O_4_) to drive the reaction and further prolonged the reaction time, but no reaction was observed (Fig. 3). In this reaction, nascent state SO_4_^2−^ should be an activated species, which generated and consumed to form SO_4_^**.–**^ very quickly in the reaction. Furthermore, we have considered the formation of persulfate (S_2_O_8_^2−^) from sulfate by TBHP (Fig. 3). We also tried to test the intermediate reaction by mixing K_2_S_2_O_8_ and TBHP under the optimized conditions. However, we found that the desired product **2a** could not be obtained. The result shows that SO_4_^**.–**^ can not be generated from S_2_O_8_^2−^ under this conditions. Besides, we tested the mass spectra. A signal at m/z = 96.9600 was observed (see Supplementary online). That was assigned to [SO_4_^2−^+H]^−^ (calculated: 96.9596) species. The results suggested that SO_4_^2−^ was only generated in the process of forming product.

On the basis of previous studies and the results of our experiments^30^,^31^, a plausible reaction mechanism of the oxidation of aromatic alcohols to aldehydes is proposed. A radical anion **A** (sulfate radical anion) could be generated by action of TBHP and Na_2_S_2_O_4_ in this reaction. **A** was found to be an excellent controllable oxidant for oxidation of aromatic alcohols **B** to aldehydes **C** under this reaction conditions.

## Conclusion

In conclusion, we have developed a new metal-free oxidation system, which was applied to oxidize various primary aromatic alcohols to corresponding aldehydes in good yields at room temperature. This is a new way to produce SO_4_^.–^ by the new oxidation system. The present protocol provides a new tool for alcohol controllable oxidation under mild conditions, and also could be used widely in general organic synthesis. Detailed mechanistic studies and other applications of the new oxidation system in organic reactions are under way in our laboratory.

## Methods

Full experimental details and characterization of the compounds can be found in the Supplementary Information.

**General**. All solvents and chemicals are used directly from commercial sources without further purification. Analytical Thin Layer Chromatography was carried out on precoated plates (silica gel 60), visualized with UV light. NMR spectra was performed on a Bruker DPX-400 spectrometer operating at 400 MHz (^1^H NMR). All spectra were recorded in CDCl_3_ and the chemical shifts (δ) are reported in ppm relative to tetramethylsilane referenced to the residual solvent peaks. High-resolution mass spectral analyses (HRMS) were measured using ESI ionization. Sulfate radical anion were determined by a DMPO spin-trapping EPR method at room temperature on a Bruker E500 spectrometer. Instrument settings were modulation frequency: 100.00 KHz; modulation amplitude: 2.00 G; sweep width: 100.00 G; time constant: 40.960 ms; conversion: 40.000 ms; sweep time: 40.96 s. The microwave power was 10.03 mW, and the frequency was 9.857 GHz.

### General Procedure for the Synthesis of Products 2

To a mixture of alcohol (0.5 mmol) and Na_2_S_2_O_4_ (174 mg, 1.0 mmol) in ethyl acetate (4 mL) was slowly added *tert*-butyl hydroperoxide (257 mg, 2.0 mmol, 70% in water). The mixture was stirred at room temperature for 12 h. After evaporation of ethyl acetate under reduced pressure, the residue was separated on a silica gel column by using petroleum ether and ethyl acetate as eluent.

## Additional Information

**How to cite this article**: Bai, C.-B. *et al.* An Unexpected Controlled New Oxidant: SO_4_^.–^. *Sci. Rep.* **6**, 20163; doi: 10.1038/srep20163 (2016).

## Supplementary Material

Supplementary Information

## Figures and Tables

**Figure 1:**
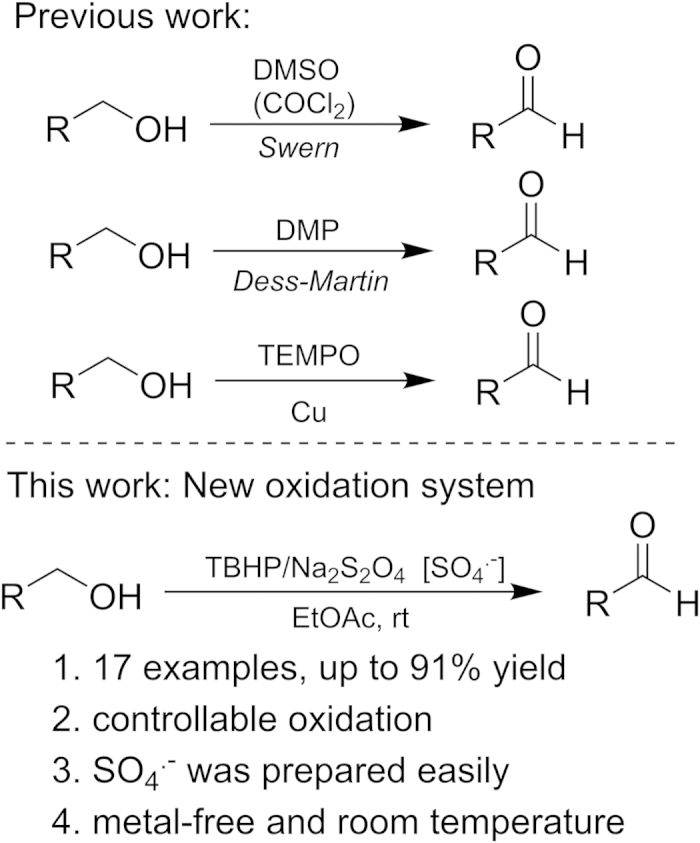
Strategies for selective oxidation of primary alcohols to aldehydes.

**Table 1:**
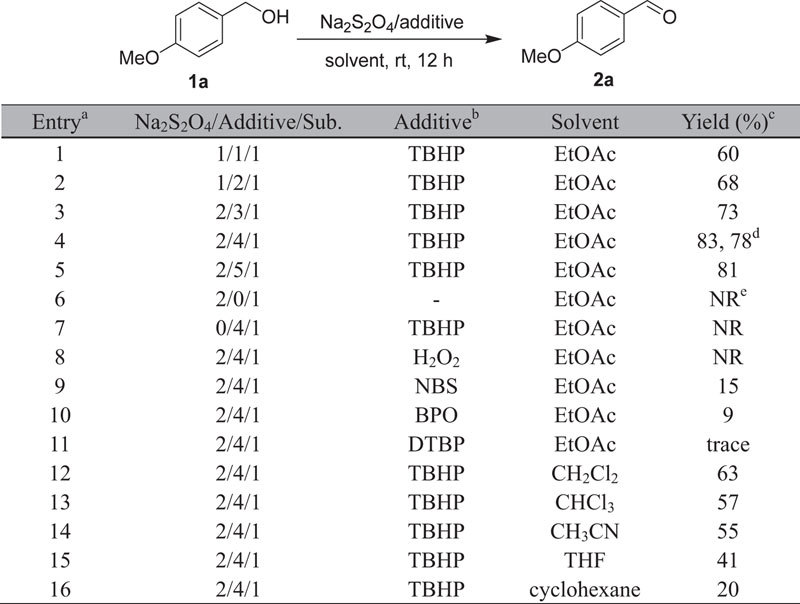
Optimization of reaction conditions.

^a^Reaction conditions: alcohol (0.5 mmol), Na_2_S_2_O_4_ and additive in solvent (4 mL) were stirred at room temperature for 12 h. ^b^TBHP = *tert*-butyl hydroperoxide, 70% = in water, DTBP = di-tert-butyl peroxide. ^c^cIsolated yield. ^d^dAnhydrous TBHP. ^e^No reaction.
