# Supplementary material for: An Unexpected Controlled New Oxidant: SO4.–
Source: Sci Rep. 2016 Feb 1;6:20163. doi: 10.1038/srep20163 (PMC4735593; doi:10.1038/srep20163)
Supplement: Supplementary Information [file srep20163-s1.doc]

An Unexpected Controlled New Oxidant: SO_4_^.-^

Cui-Bing Bai^1^, Nai-Xing Wang^1*^, Xing-Wang Lan^1^, Yan-Jing Wang^1^, Yalan Xing^2*^, Jia-Long Wen^1^, Xue-Wang Gao^1^ & Wei Zhang^1^

^1^Technical Institute of Physics and Chemistry, Chinese Academy of Sciences, Beijing, 100190, China

^2^Department of Chemistry, William Paterson University of New Jersey, 300 Pompton Road, Wayne, New Jersey 07470, United States

Fax: +86-10-62554670; Tel: +86-10-82543575 [nxwang@mail.ipc.ac.cn](mailto:nxwang@mail.ipc.ac.cn)

Table of Contents:

[1. Experimental section 1](#__RefHeading___Toc31847)

[2. Analytical data for **2a** – **2q** 1](#__RefHeading___Toc449)

[3. ^1^H and ^13^C NMR spectra for **2a** – **2q** 4](#__RefHeading___Toc5188)

[4. HRMS spectra 38](#__RefHeading___Toc25009)

[5. EPR spectra (Figure S1 and S2) 41](#__RefHeading___Toc25721)

1. **Experimental section**

**General:** All solvents and chemicals are used directly from commercial sources without further purification. Analytical Thin Layer Chromatography was carried out on precoated plates (silica gel 60), visualized with UV light. NMR spectra was performed on a Bruker DPX-400 spectrometer operating at 400 MHz (^1^H NMR). All spectra were recorded in CDCl_3_ and the chemical shifts (δ) are reported in ppm relative to tetramethylsilane referenced to the residual solvent peaks. High-resolution mass spectral analyses (HRMS) were measured using ESI ionization. Sulfate radical anion were determined by a DMPO spin-trapping EPR method at room temperature on a Bruker E500 spectrometer. Instrument settings were modulation frequency: 100.00 KHz; modulation amplitude: 2.00 G; sweep width: 100.00 G; time constant: 40.960 ms; conversion: 40.000 ms; sweep time: 40.96 s. The microwave power was 10.03 mW, and the frequency was 9.857 GHz.

*General Procedure for the Synthesis of Products* ***2***

To a mixture of alcohol (0.5 mmol) and Na_2_S_2_O_4_ (174 mg, 1.0 mmol) in ethyl acetate (4 mL) was slowly added *tert*-butyl hydroperoxide (257 mg, 2.0 mmol, 70% in water). The mixture was stirred at room temperature for 12 h. After evaporation of ethyl acetate under reduced pressure, the residue was separated on a silica gel column by using petroleum ether and ethyl acetate as eluent.

1. Analytical data for **2a** – **2q**

*4-methoxybenzaldehyde (****2a****).*^1^ Colorless oil; ^1^H NMR (400 MHz, CDCl_3_) δ 9.81 (s, 1H), 7.76 (d, *J* = 8.73 Hz, 2H), 6.93 (d, *J* = 8.74 Hz, 2H), 3.81 (s, 3H); ^13^C NMR (100 MHz, CDCl_3_) δ 190.77 , 164.61 , 131.95, 129.95, 114.31, 55.56; HRMS (ESI) *m/z*: [M+H]^+^ calcd for C_8_H_8_O_2_ 137.0597; found, 137.0594.

*3-methoxybenzaldehyde (****2b****).* Colorless oil; ^1^H NMR (400 MHz, CDCl_3_) δ 10.51 (s, 1H), 7.86 (dd, *J* = 7.68, 1.78 Hz, 1H), 7.62-7.56 (m, 1H), 7.10-7.00 (m, 2H), 3.96 (s, 3H); ^13^C NMR (100 MHz, CDCl_3_) δ 192.12, 160.15, 137.81, 130.02, 123.44, 121.43, 112.15, 55.42.

*2-methoxybenzaldehyde (****2c****).* Colorless oil; ^1^H NMR (400 MHz, CDCl_3_) δ 10.01 (s, 1H), 7.50-7.48 (m, 2H), 7.43 (d, *J* = 2.22 Hz, 1H), 7.23-7.20 (m, 1H), 3.90 (s, 3H); ^13^C NMR (100 MHz, CDCl_3_) δ 189.85, 161.85, 135.95, 128.58, 124.86, 120.68, 111.63, 55.64.

*4-methylbenzaldehyde (****2d****).*^1^ Colorless oil; ^1^H NMR (400 MHz, CDCl_3_) δ 9.98 (s, 1H), 7.79 (d, *J* = 8.03 Hz, 2H), 7.34 (d, *J* = 7.93 Hz, 2H), 2.45 (s, 3H); ^13^C NMR (100 MHz, CDCl_3_) δ 191.99, 145.55 , 134.21, 129.84, 129.71, 21.86.

*benzaldehyde (****2e****).* Colorless oil; ^1^H NMR (400 MHz, CDCl_3_) δ 10.02 (s, 1H), 7.88 (d, *J* = 7.16 Hz, 2H), 7.63 (d, *J* = 7.37 Hz, 1H), 7.53 (d, *J* = 7.60 Hz, 2H); ^13^C NMR (100 MHz, CDCl_3_) δ 192.38, 136.42, 134.46, 129.73, 129.00.

*4-chlorobenzaldehyde (****2f****).*^1^ White solid; ^1^H NMR (400 MHz, CDCl_3_) δ 10.01 (s, 1H), 7.85 (d, *J* = 8.38 Hz, 2H), 7.54 (d, *J* = 8.37 Hz, 2H); ^13^C NMR (100 MHz, CDCl_3_) δ 190.85, 140.96, 134.73, 130.91, 129.46.

*4-bromobenzaldehyde (****2g****).*^1^ White solid; ^1^H NMR (400 MHz, CDCl_3_) δ 10.00 (s, 1H), 7.78-7.76 (m, 2H), 7.73-7.70 (m, 2H); ^13^C NMR (100 MHz, CDCl_3_) δ 191.06, 135.09, 132.45, 130.98, 129.79.

*4-nitrobenzaldehyde* *(****2h****).*^1^ Yellow solid; ^1^H NMR (400 MHz, CDCl_3_) δ 10.10 (s, 1H), 8.33 (d, *J* = 8.63 Hz, 2H), 8.01 (d, *J* = 8.69 Hz, 2H); ^13^C NMR (100 MHz, CDCl_3_) δ 190.27, 151.16, 140.08, 130.48 , 124.31.

*3-nitrobenzaldehyde* *(****2i****).* Yellow solid; ^1^H NMR (400 MHz, CDCl_3_) δ 10.15 (s, 1H), 8.74 (s, 1H), 8.52 (d, *J* = 8.12 Hz, 1H), 8.26 (d, *J* = 7.62 Hz, 1H), 7.80 (d, *J* = 7.90 Hz, 1H); ^13^C NMR (100 MHz, CDCl_3_) δ 189.71, 148.81, 137.41, 134.62, 130.39, 128.60, 124.50.

*2-nitrobenzaldehyde* *(****2j****).*^1^ Yellow solid; ^1^H NMR (400 MHz, CDCl_3_) δ 10.45 (s, 1H), 8.15 (d, *J* = 7.77 Hz, 1H), 7.99 (d, *J* = 7.47 Hz, 1H), 7.85-7.77 (m, 2H); ^13^C NMR (100 MHz, CDCl_3_) δ 188.12, 149.63, 134.07, 133.70, 131.37, 129.64, 124.50.

*1-naphthaldehyde (****2k****).*^1^ Yellow solid; ^1^H NMR (400 MHz, CDCl_3_) δ 10.43 (s, 1H), 9.29 (d, *J* = 8.58 Hz, 1H), 8.12 (d, *J* = 8.24 Hz, 1H), 8.01 (d, *J* = 7.04 Hz, 1H), 7.95 (d, *J* = 8.17 Hz, 1H), 7.75-7.70 (m, 1H), 7.67-7.61 (m, 2H); ^13^C NMR (100 MHz, CDCl_3_) δ 193.55, 136.67, 135.30, 133.75, 131.43, 130.55, 129.08, 128.50, 126.98, 124.89.

*2-naphthaldehyde (****2l****).* Yellow solid; ^1^H NMR (400 MHz, CDCl_3_) δ 10.19 (s, 1H), 8.37 (s, 1H), 8.05-7.93 (m, 4H), 7.70-7.60 (m, 2H); ^13^C NMR (100 MHz, CDCl_3_) δ 192.28, 136.46, 134.58, 134.12, 132.65, 129.55, 129.14, 129.11, 128.10, 127.11, 122.77; HRMS (ESI) *m/z*: [M+H]^+^ calcd for C_11_H_8_O 157.0648; found, 157.0641.

*furan-2-carbaldehyde (****2m****).*^1^ Yellow oil; ^1^H NMR (400 MHz, CDCl_3_) δ 9.60 (s, 1H), 7.63 (d, *J* = 0.46 Hz, 1H), 7.19 (d, *J* = 3.59 Hz, 1H), 6.54 (dd, *J* = 3.53, 1.56 Hz, 1H); ^13^C NMR (100 MHz, CDCl_3_) δ 177.85, 152.92, 148.11, 121.17, 112.59.

*thiophene-2-carbaldehyde (****2n****).*^1^ Yellow oil; ^1^H NMR (400 MHz, CDCl_3_) δ 9.97 (s, 1H), 7.80 (dd, *J* = 7.18, 4.35 Hz, 2H), 7.24 (t, *J* = 4.32 Hz, 1H); ^13^C NMR (100 MHz, CDCl_3_) δ 183.05, 143.98, 136.50, 135.17, 128.41; HRMS (ESI) *m/z*: [M+H]^+^ calcd for C_5_H_4_OS 113.0056; found, 113.0052.

*picolinaldehyde (****2o****).* Colorless oil; ^1^H NMR (400 MHz, CDCl_3_) δ 10.07 (s, 1H), 8.78 (d, *J* = 3.73 Hz, 1H), 7.95 (d, *J* = 7.73 Hz, 1H), 7.87 (d, *J* = 7.62 Hz, 1H), 7.52 (d, *J* = 6.06 Hz, 1H); ^13^C NMR (100 MHz, CDCl_3_) δ 193.34, 152.73, 150.16, 137.02, 127.83, 121.64.

*3,4-dimethoxybenzaldehyde (****2p****).*^1^ White solid; ^1^H NMR (400 MHz, CDCl_3_) δ 9.89 (s, 1H), 7.50-7.47 (m, 1H), 7.44 (s, 1H), 7.01 (dd, *J* = 8.15, 3.41 Hz, 1H), 4.00 (s, 3H), 3.97 (s, 3H); ^13^C NMR (100 MHz, CDCl_3_) δ 190.88, 154.52, 149.66, 130.18, 126.86, 110.42, 108.99, 56.19, 56.02; HRMS (ESI) *m/z*: [M+H]^+^ calcd for C_9_H_10_O_3_ 167.0703; found, 167.0706.

*3,4,5-trimethoxybenzaldehyde (****2q****).* White solid; ^1^H NMR (400 MHz, CDCl_3_) δ 9.89 (s, 1H), 7.15 (s, 2H), 3.96 (d, *J* = 3.06 Hz, 9H); ^13^C NMR (100 MHz, CDCl_3_) δ 191.04, 153.67, 143.67, 131.74, 106.76, 61.00, 56.30; HRMS (ESI) *m/z*: [M+H]^+^ calcd for C_10_H_12_O_4_ 197.0808; found, 197.0809.

1. Liu, X.-L.; Xia, Q.-Q.; Zhang, Y.-J.; Chen, C.-Y.; Chen, W.-Z. *J. Org. Chem.* **2013**, *78*, 8531.

**3.** ^1^H and ^13^C NMR spectra for **2a – 2q**


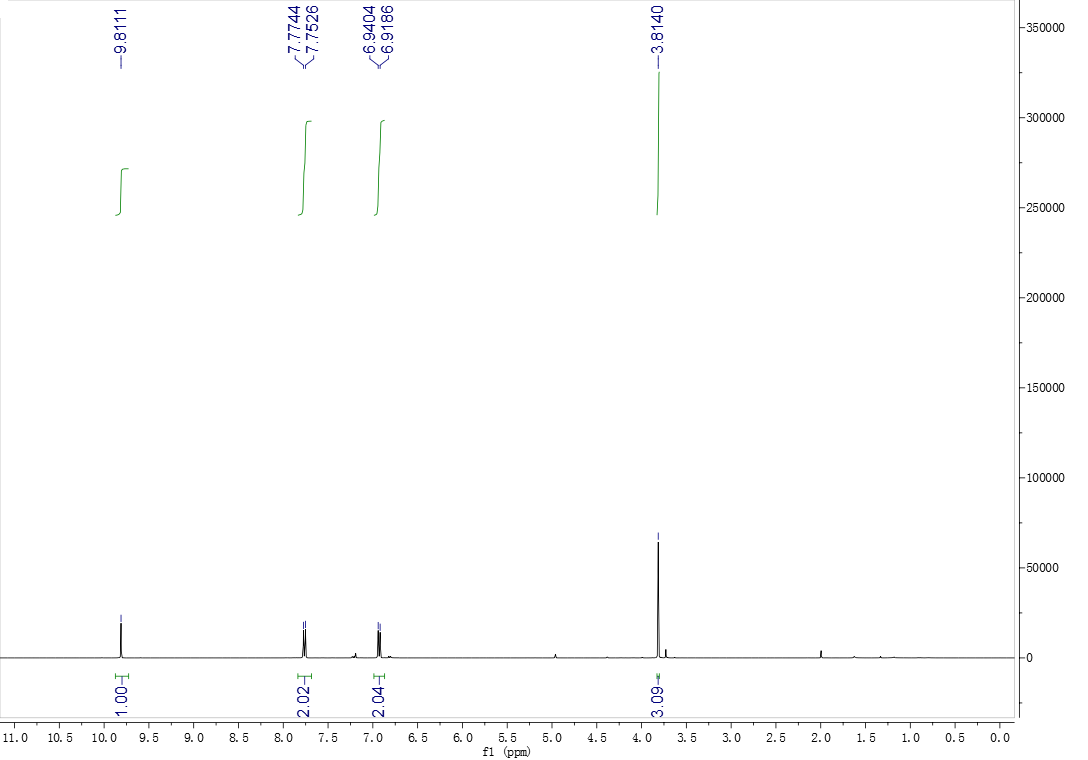


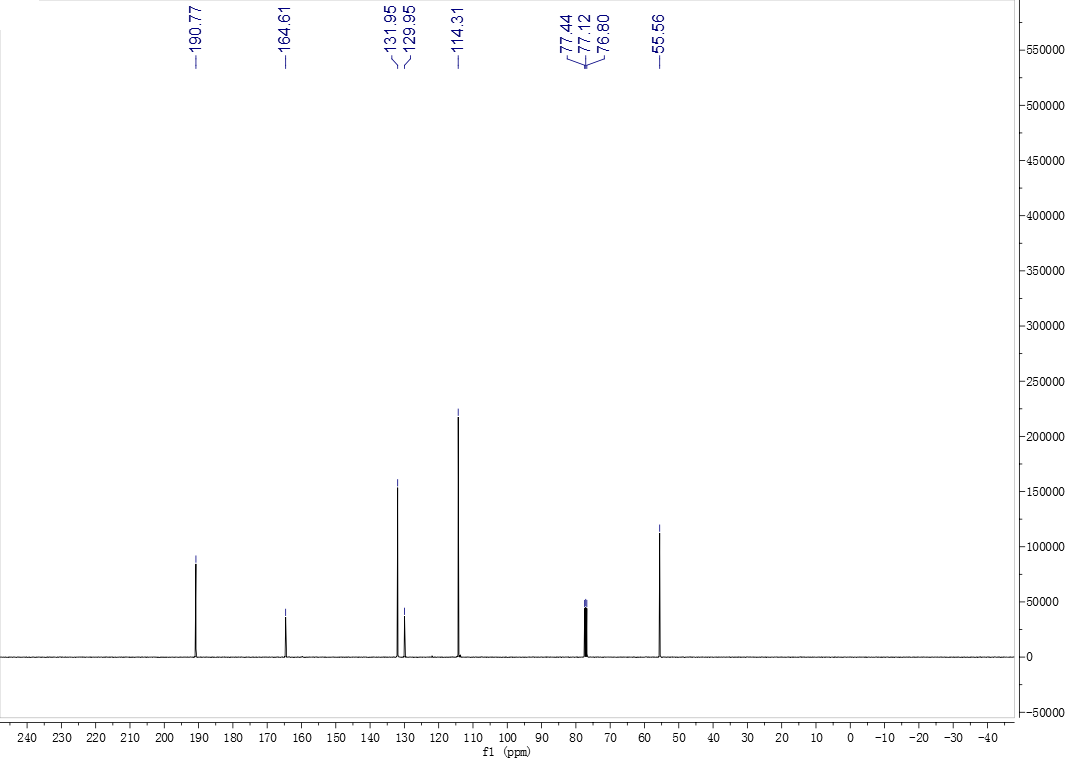


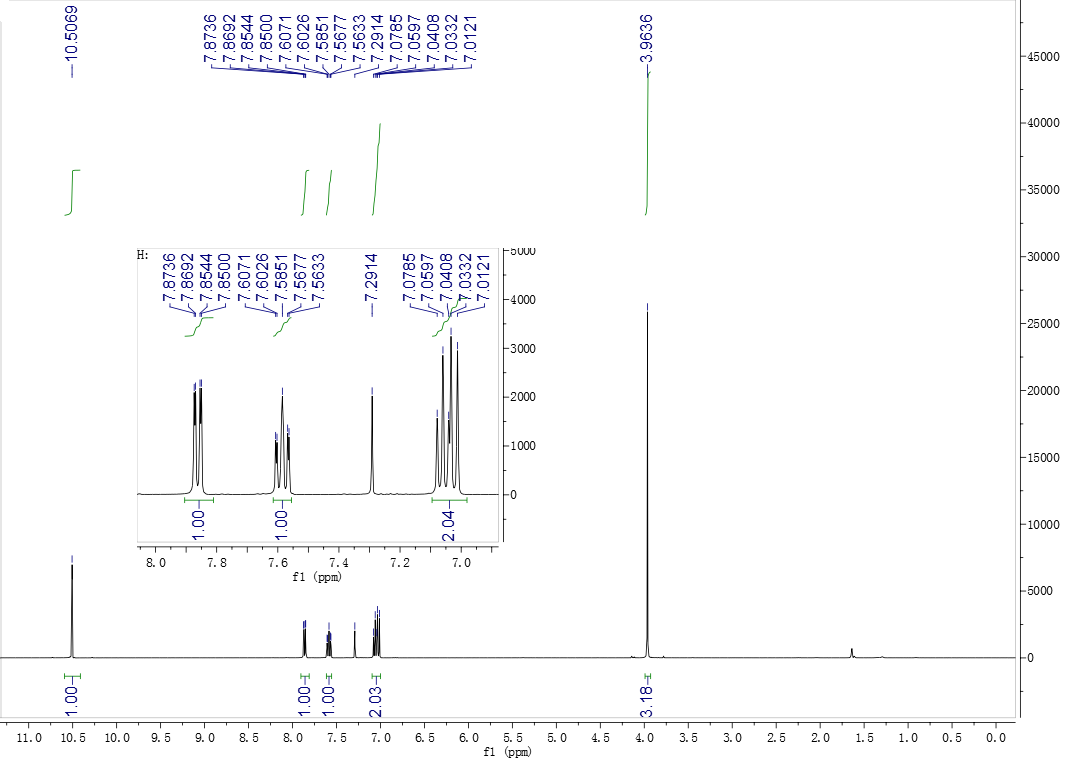


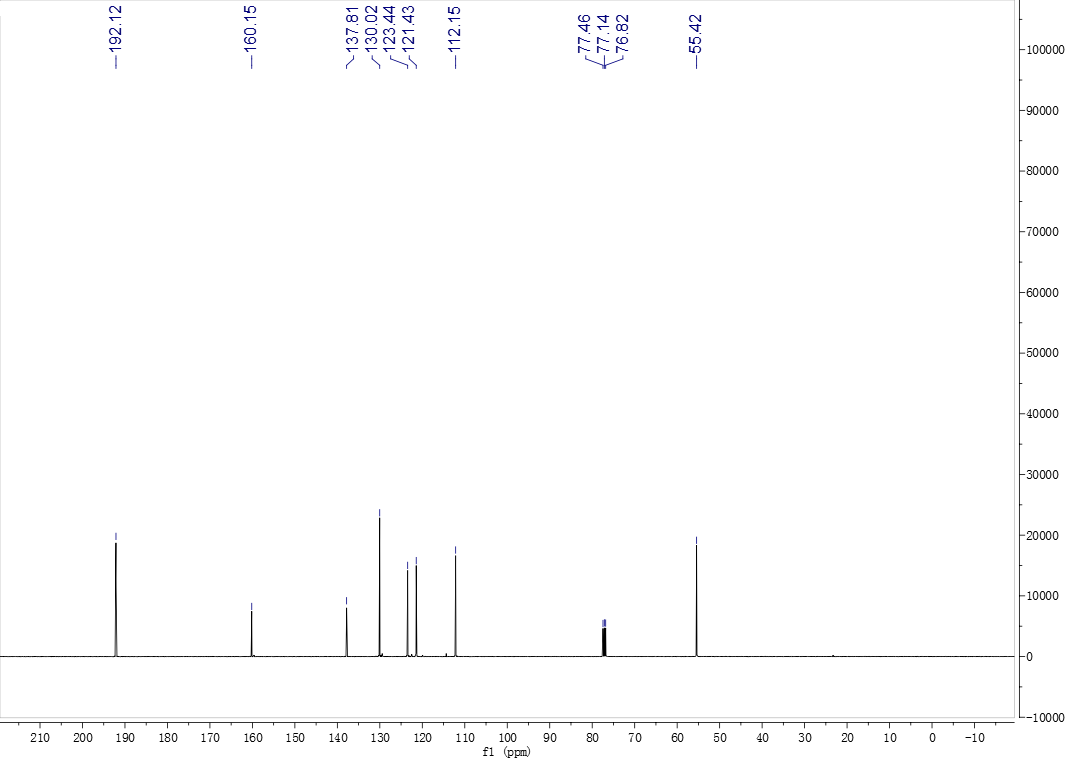


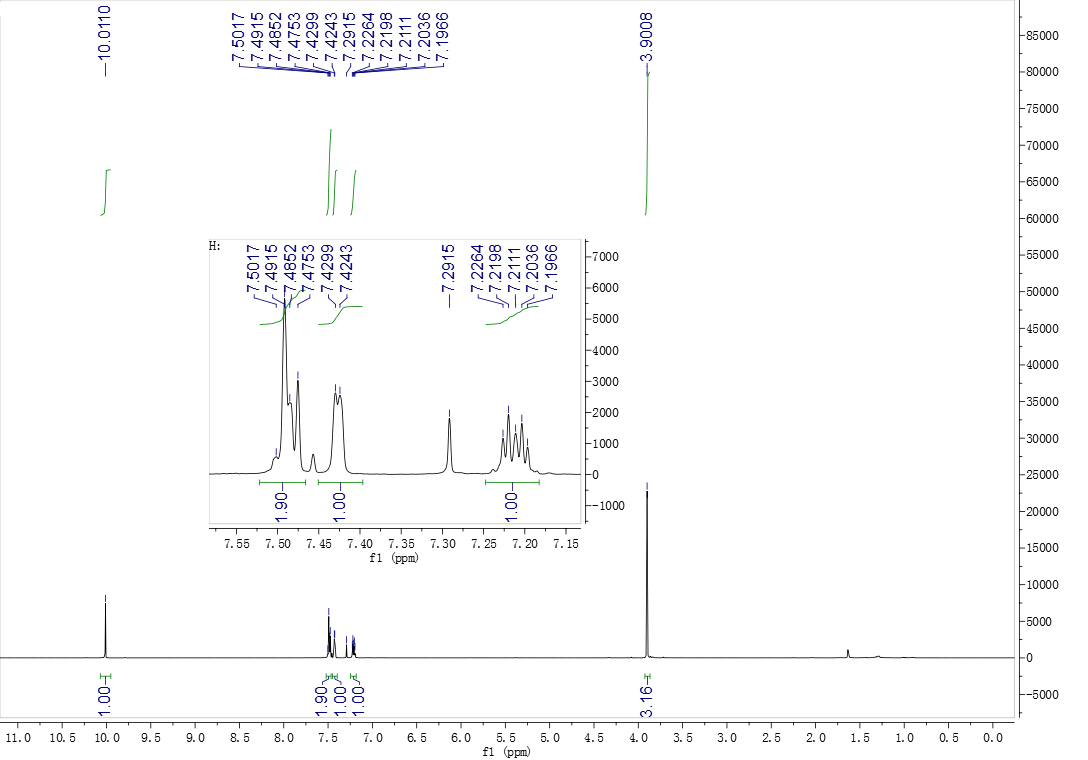


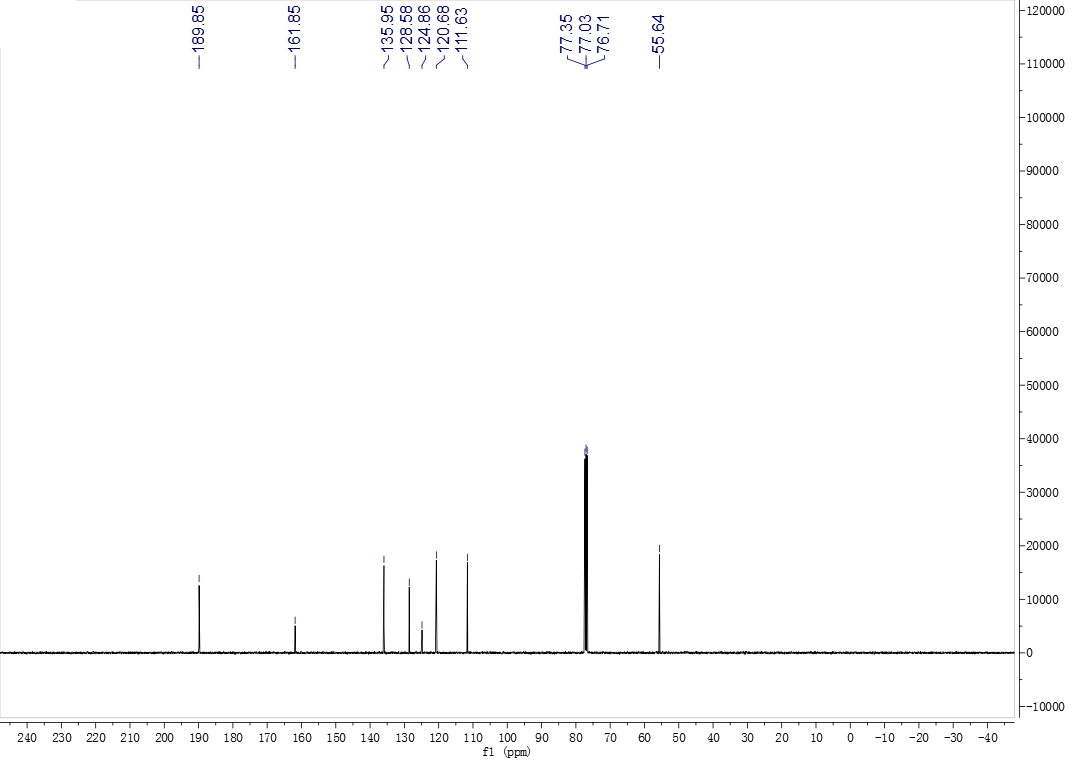


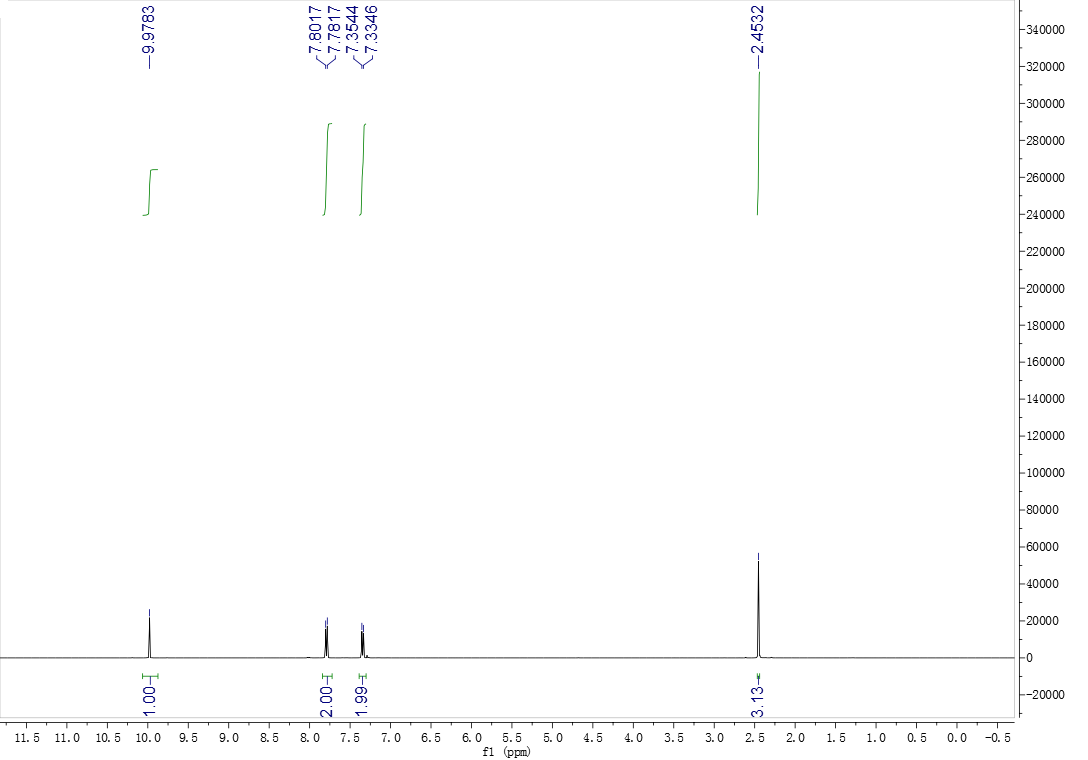


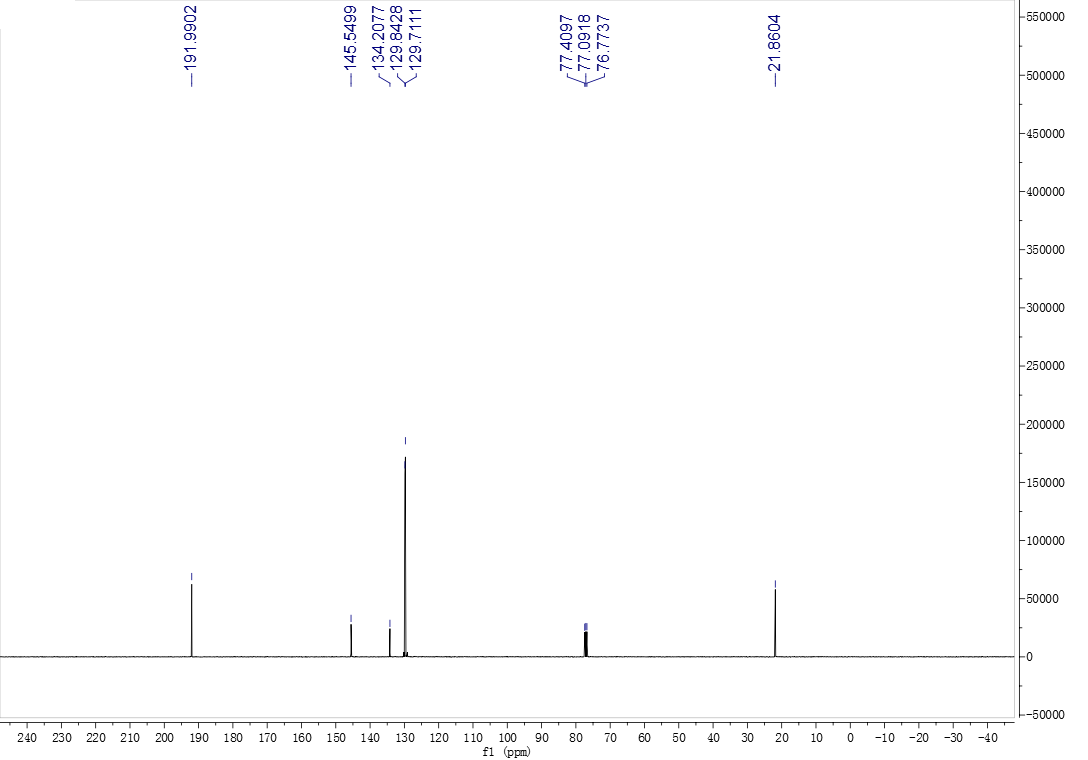


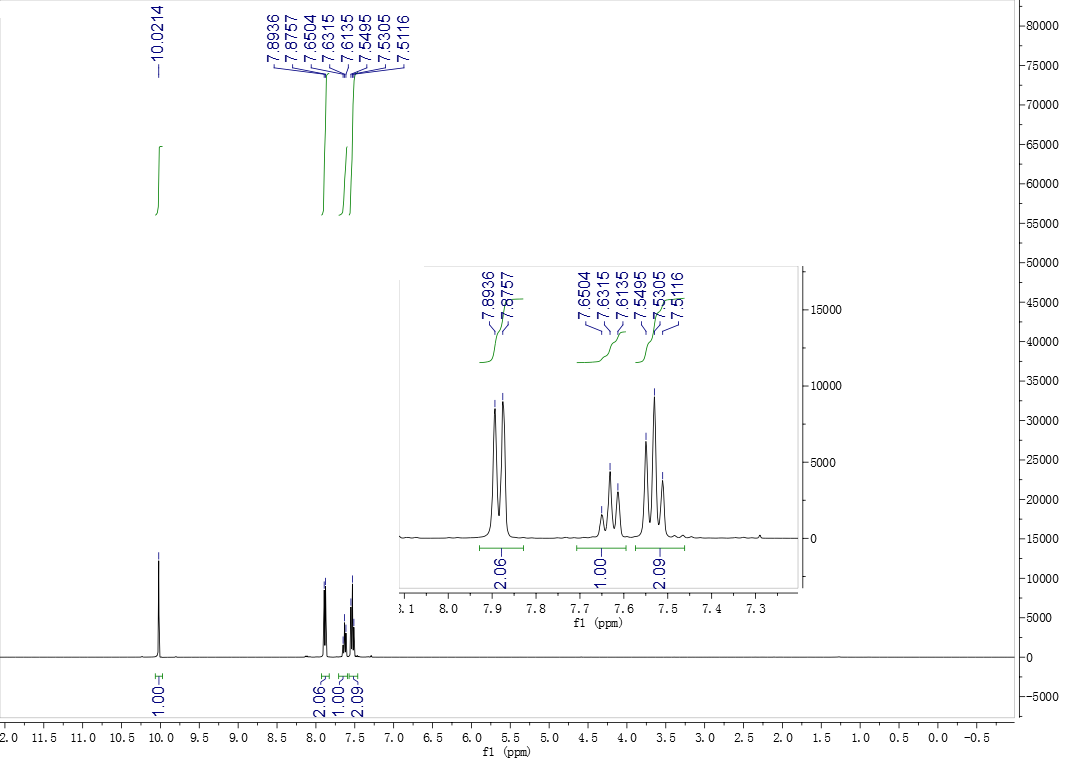


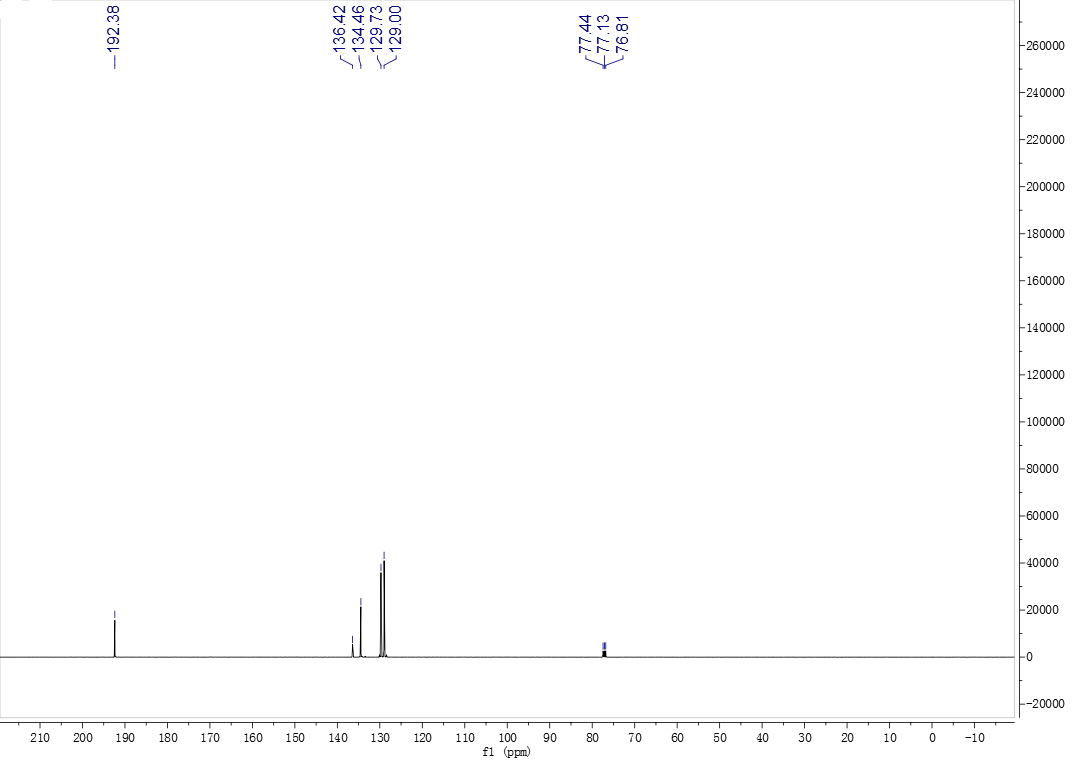


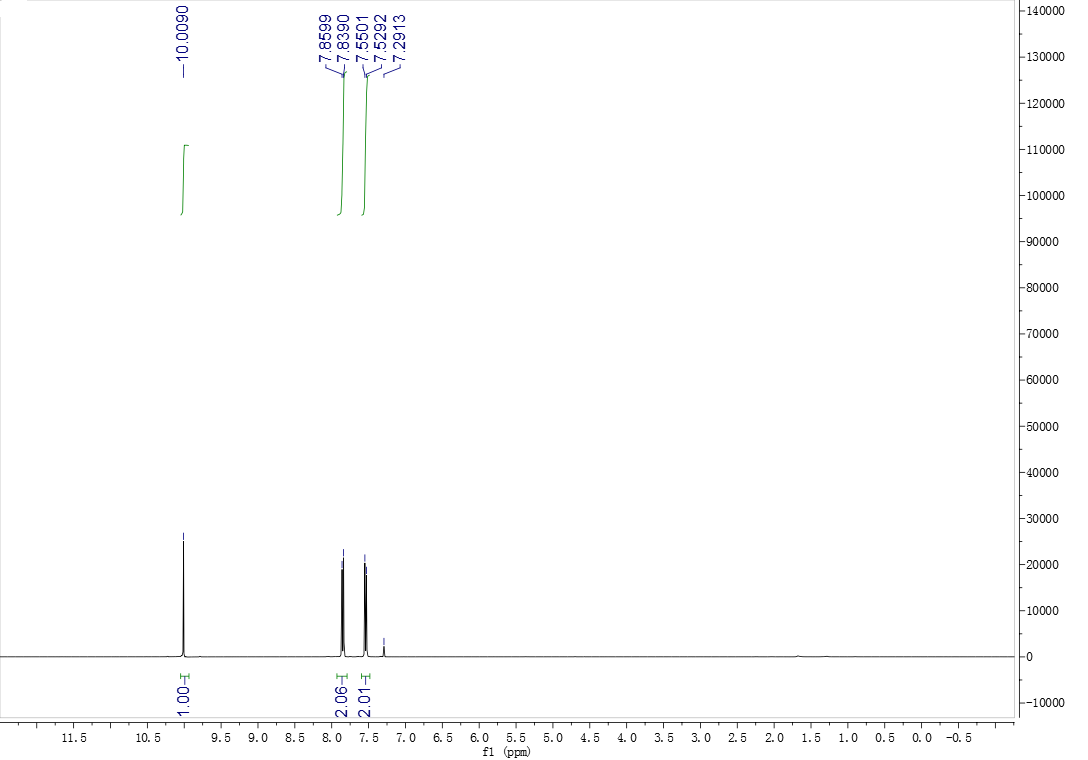


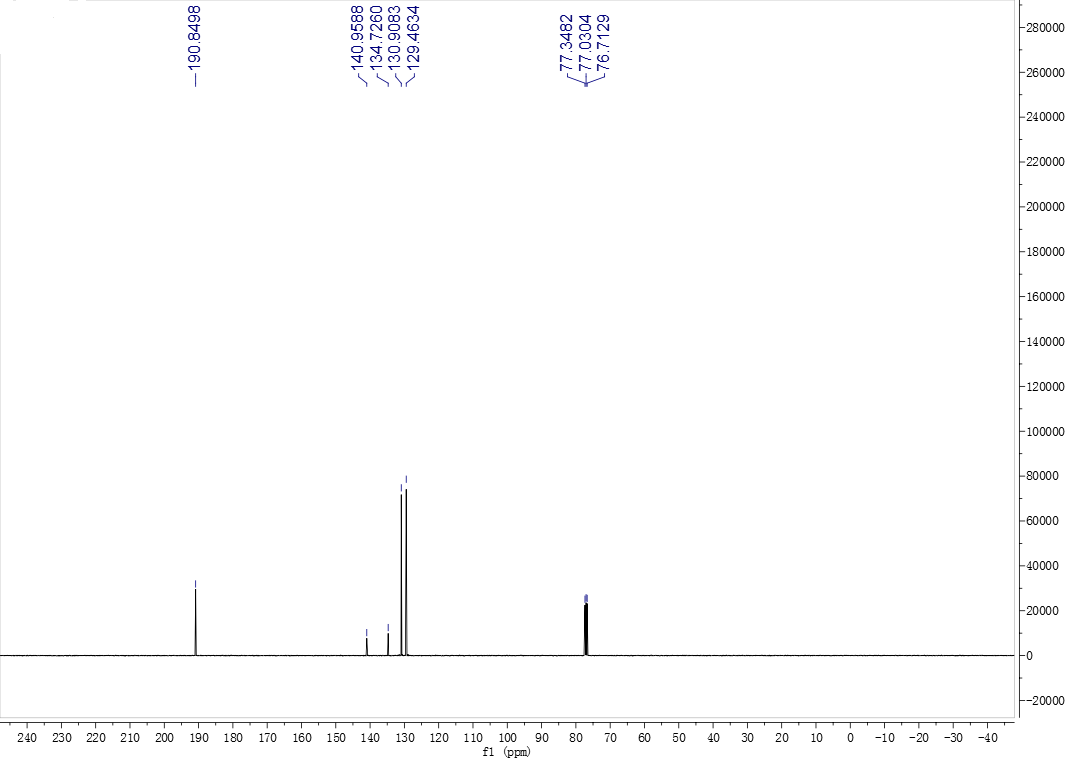


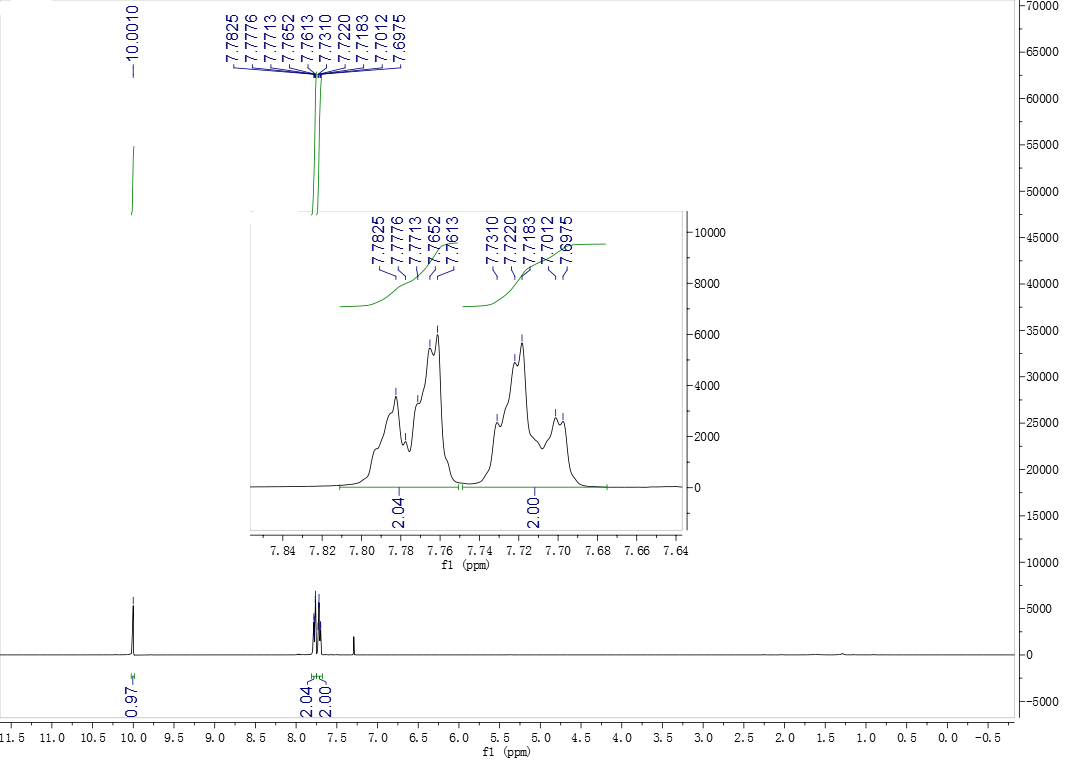


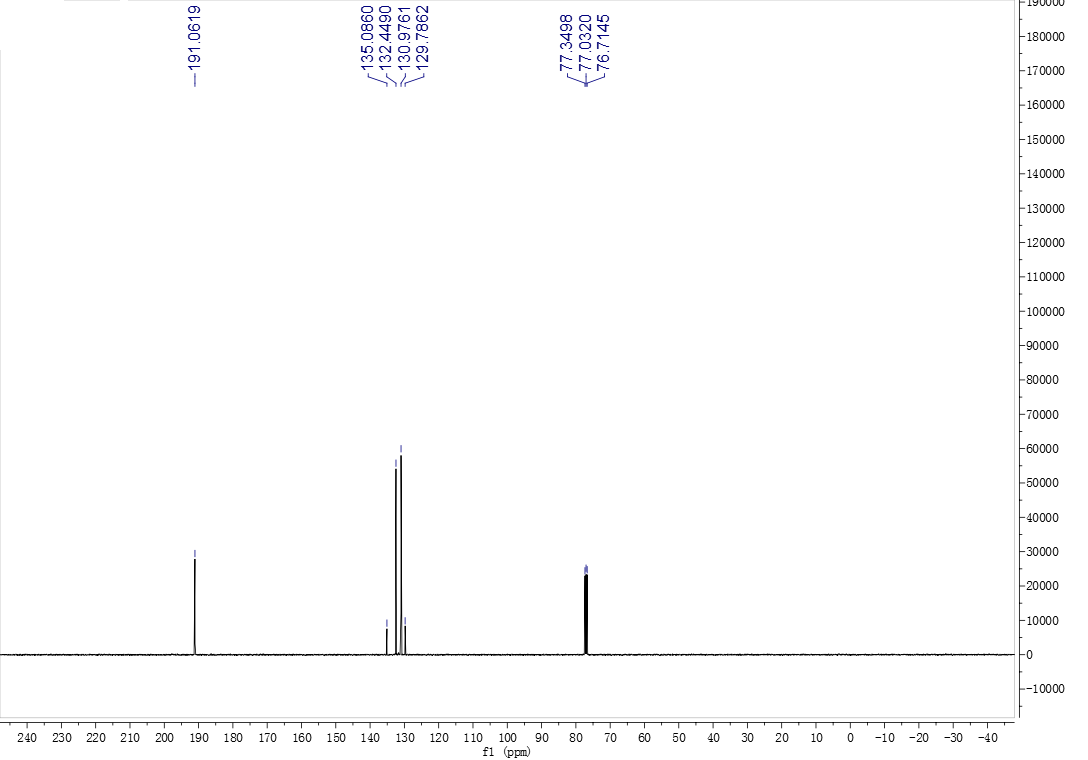


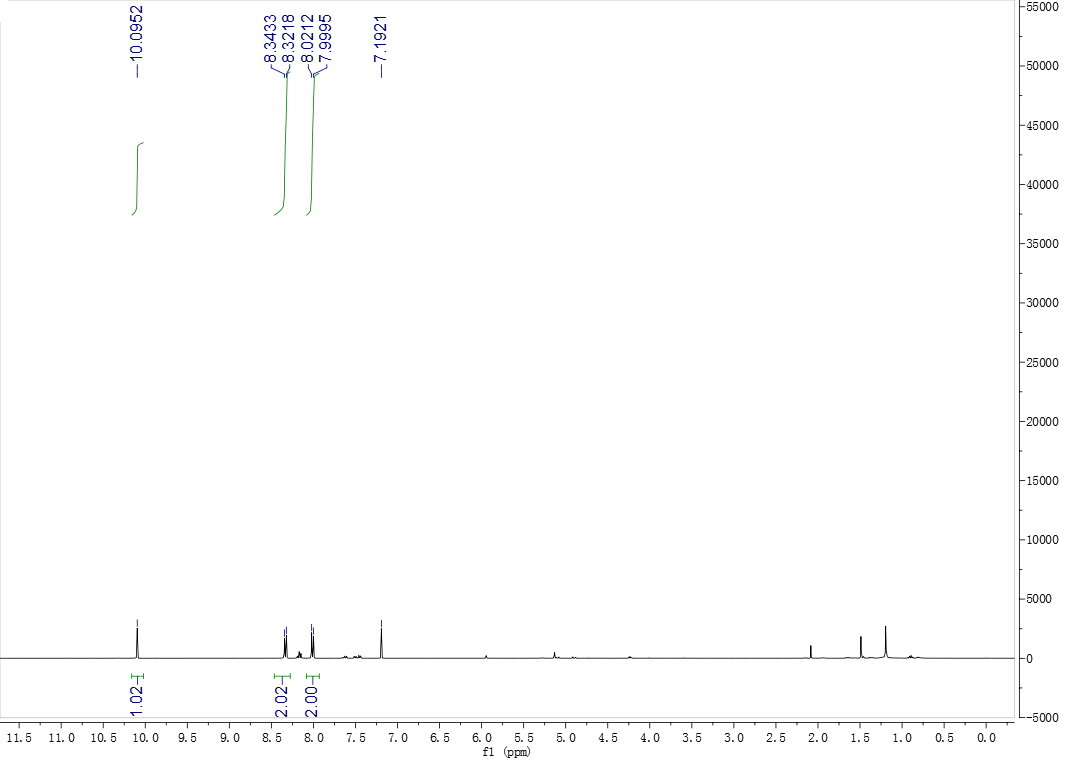


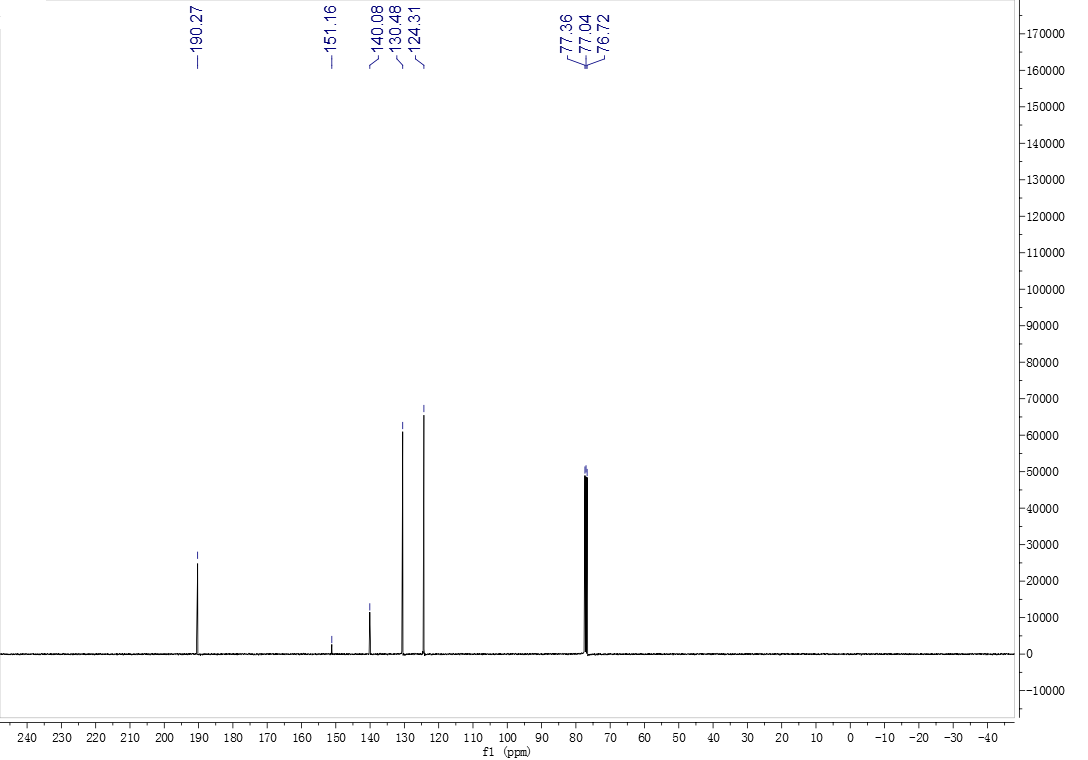


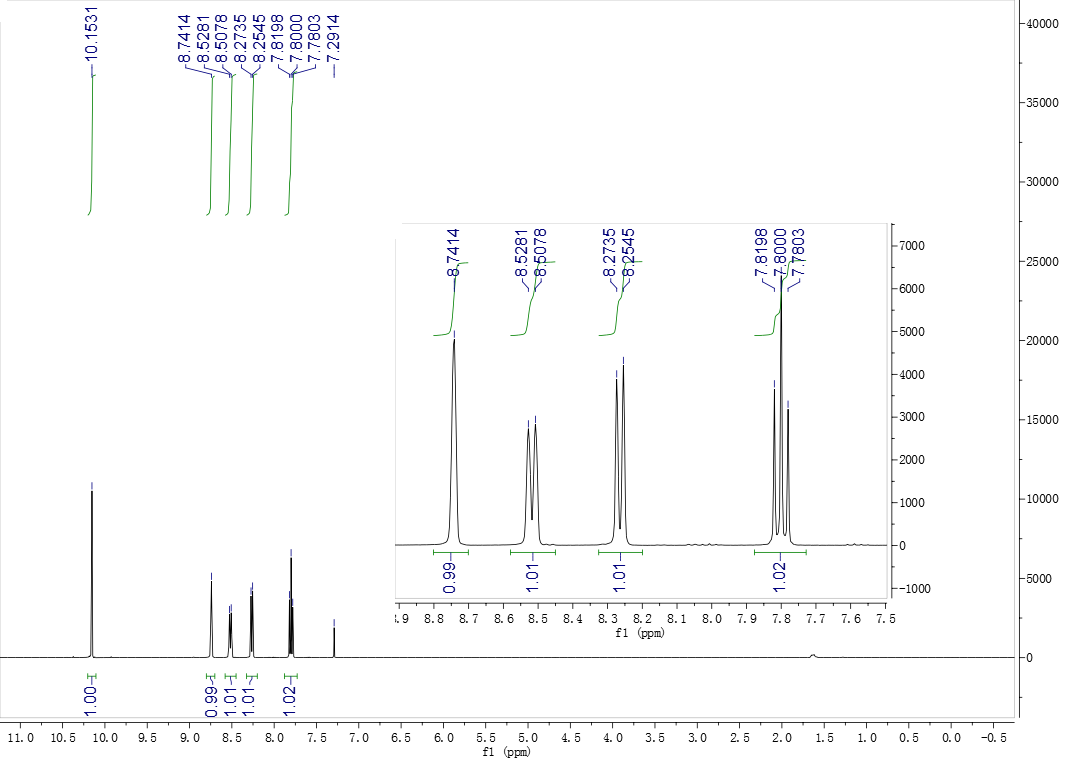


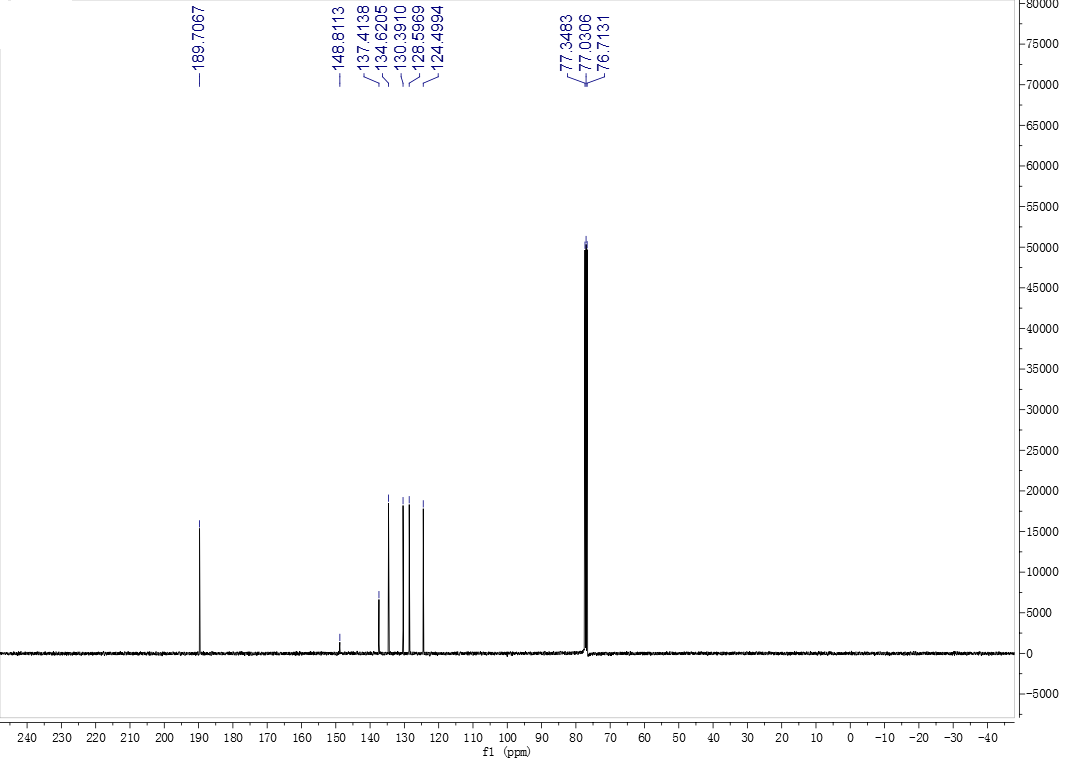


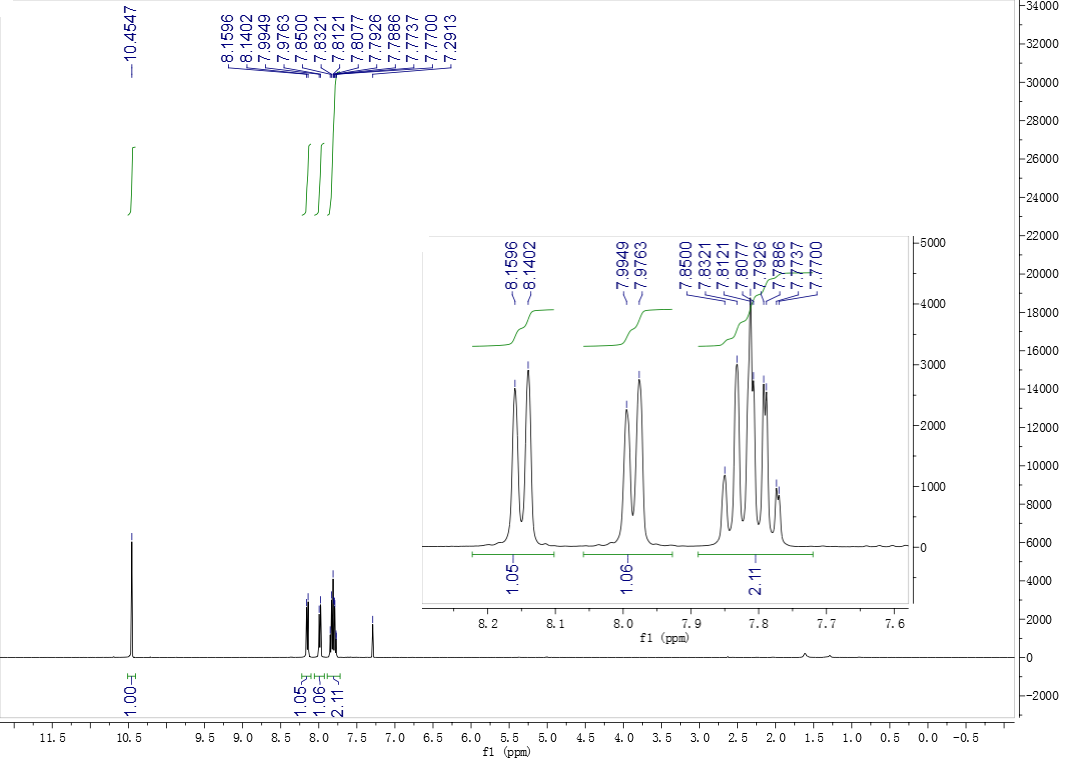


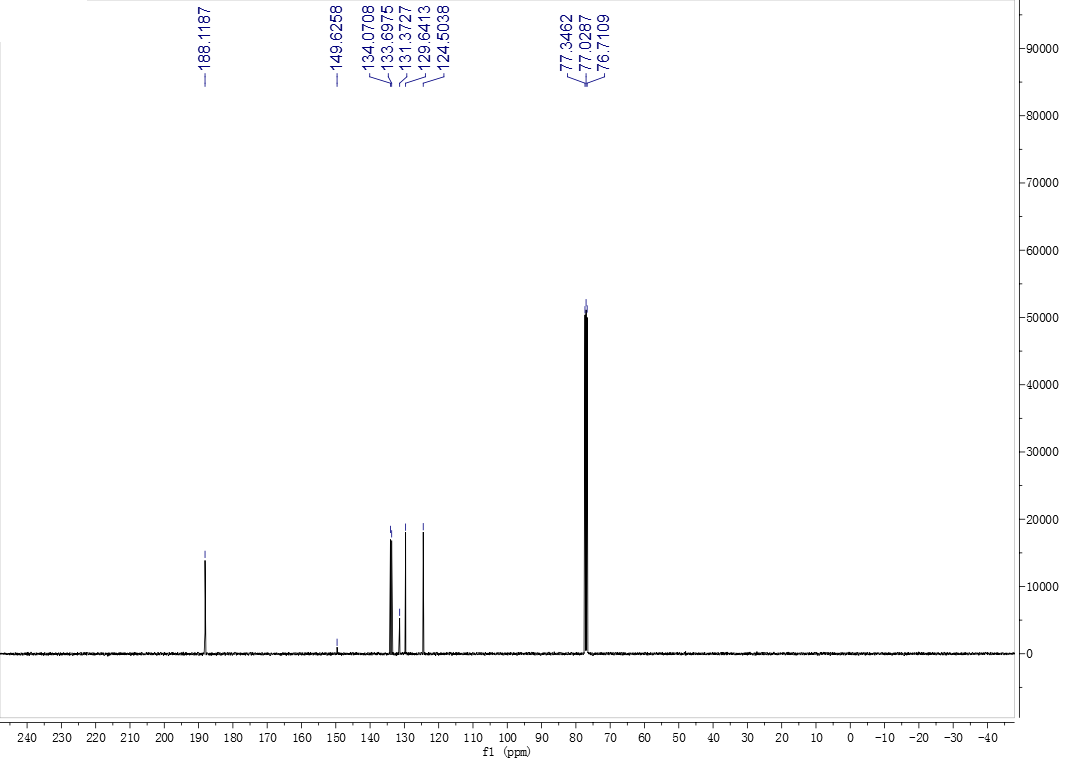


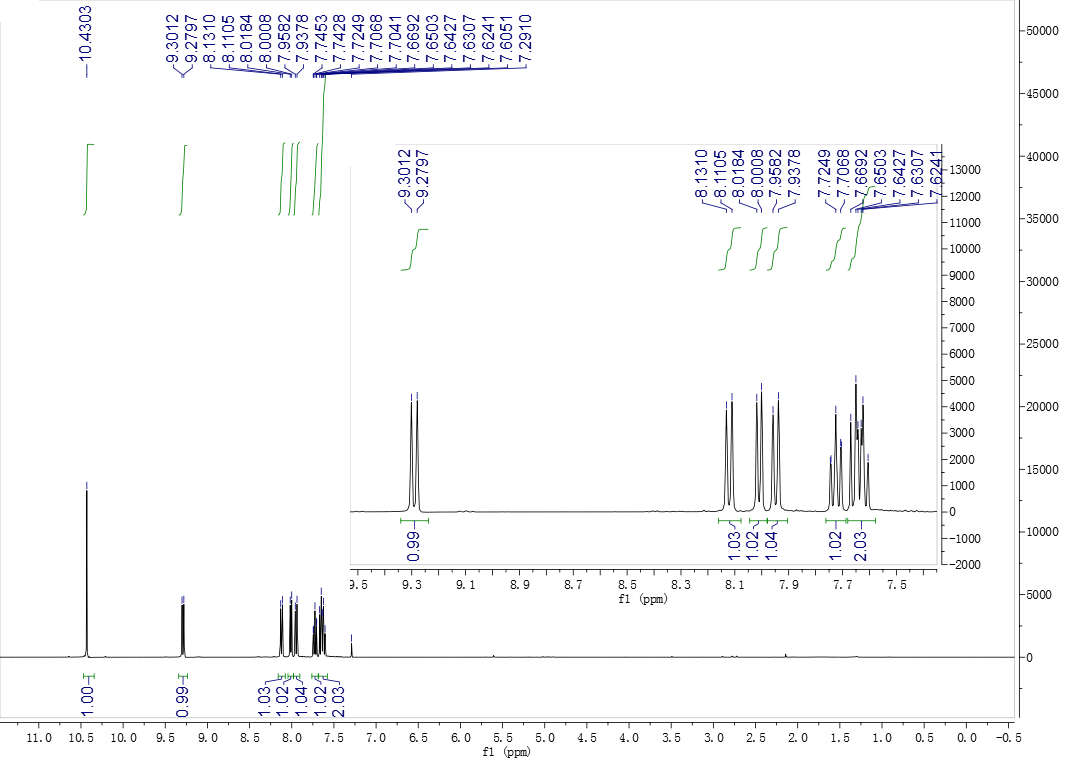


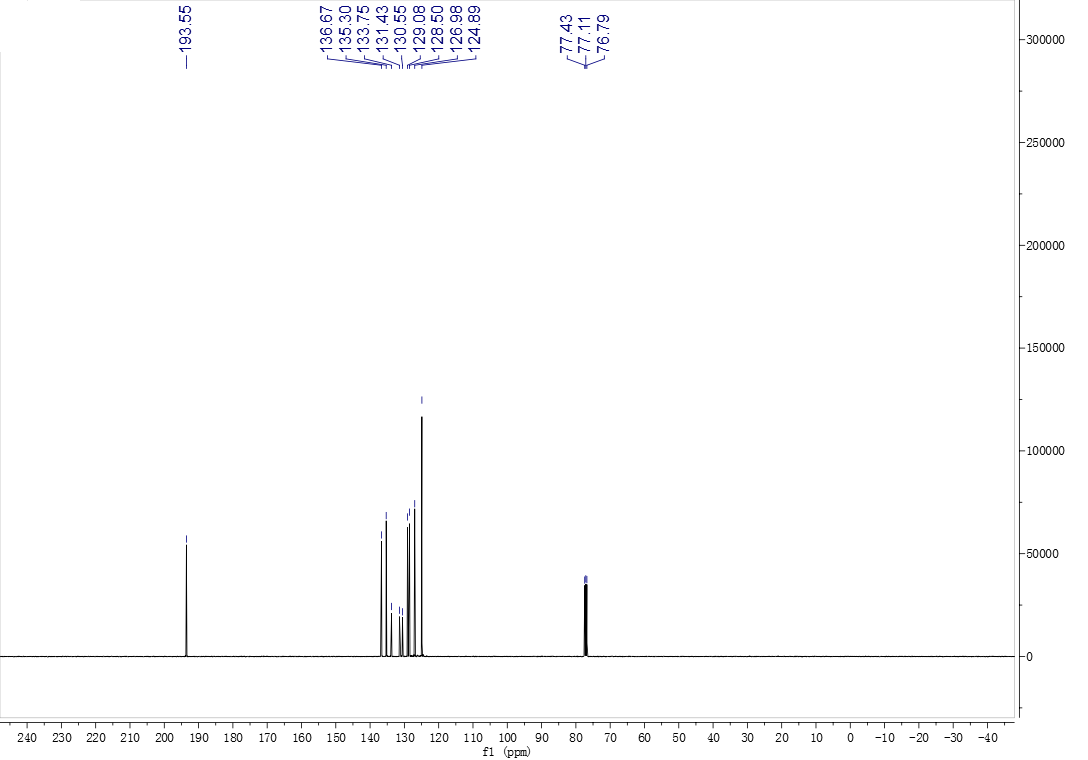


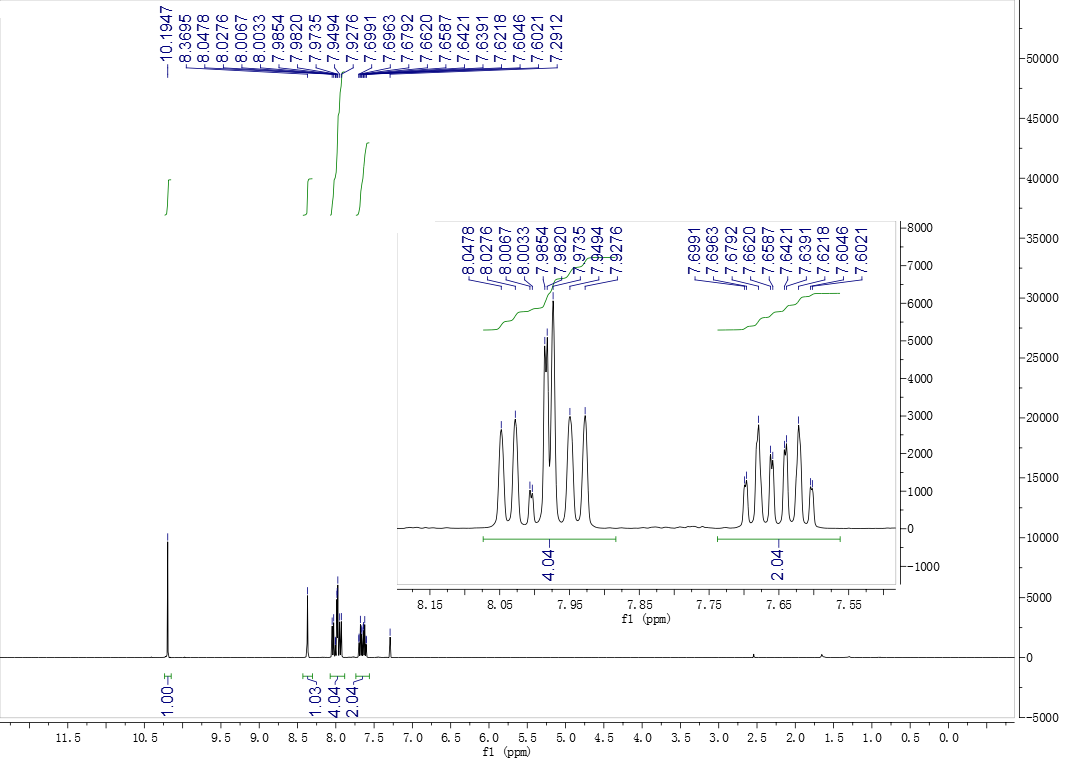


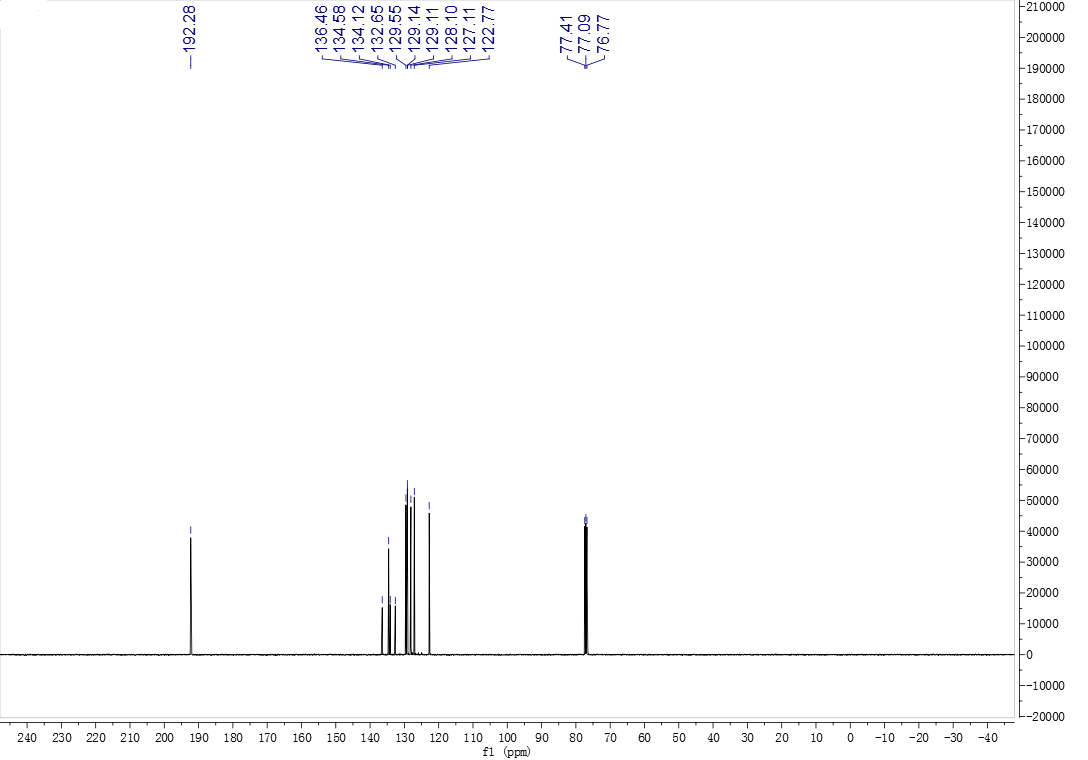


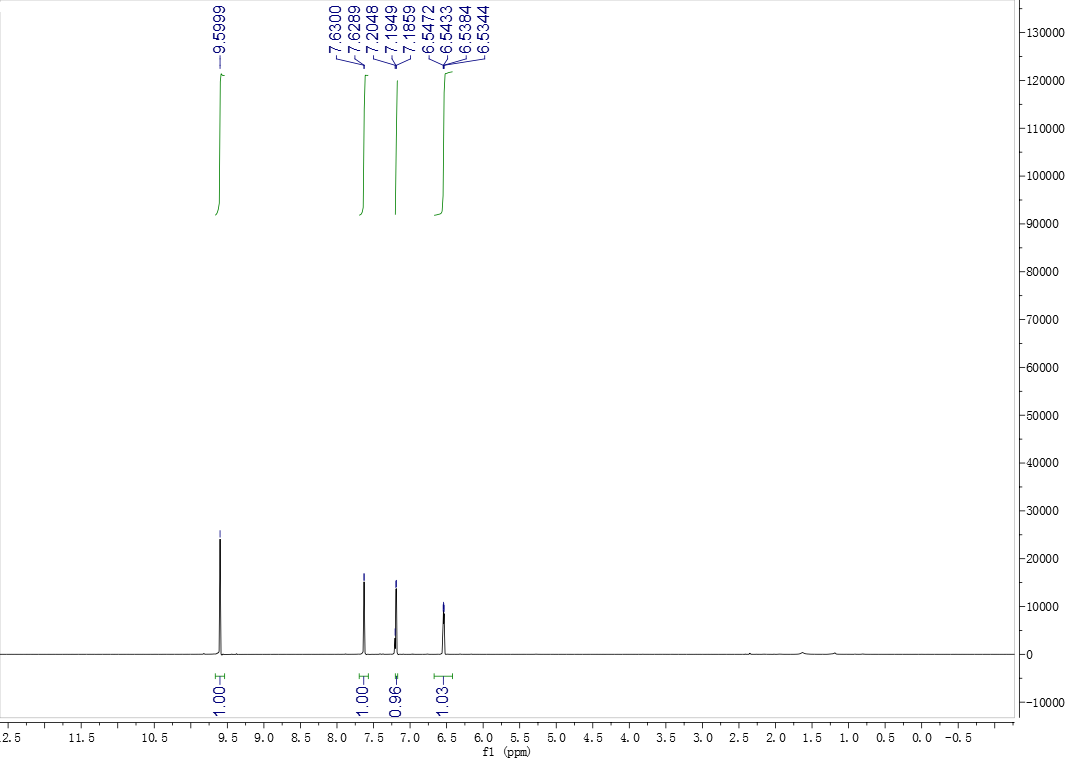


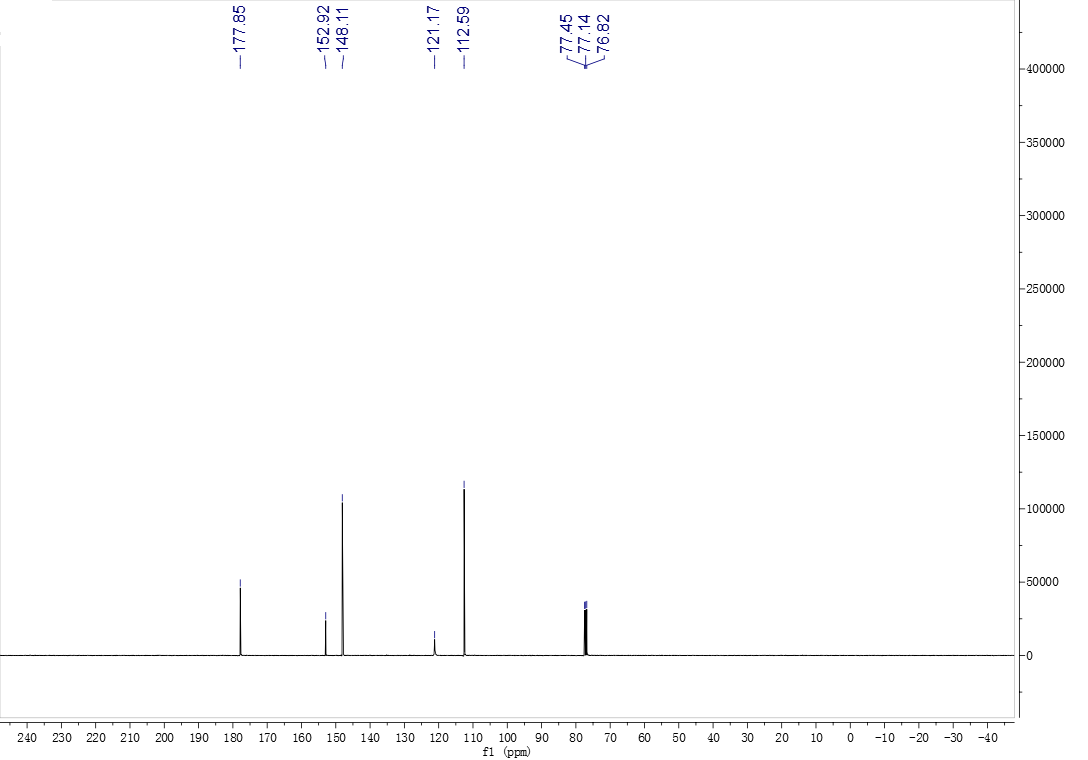


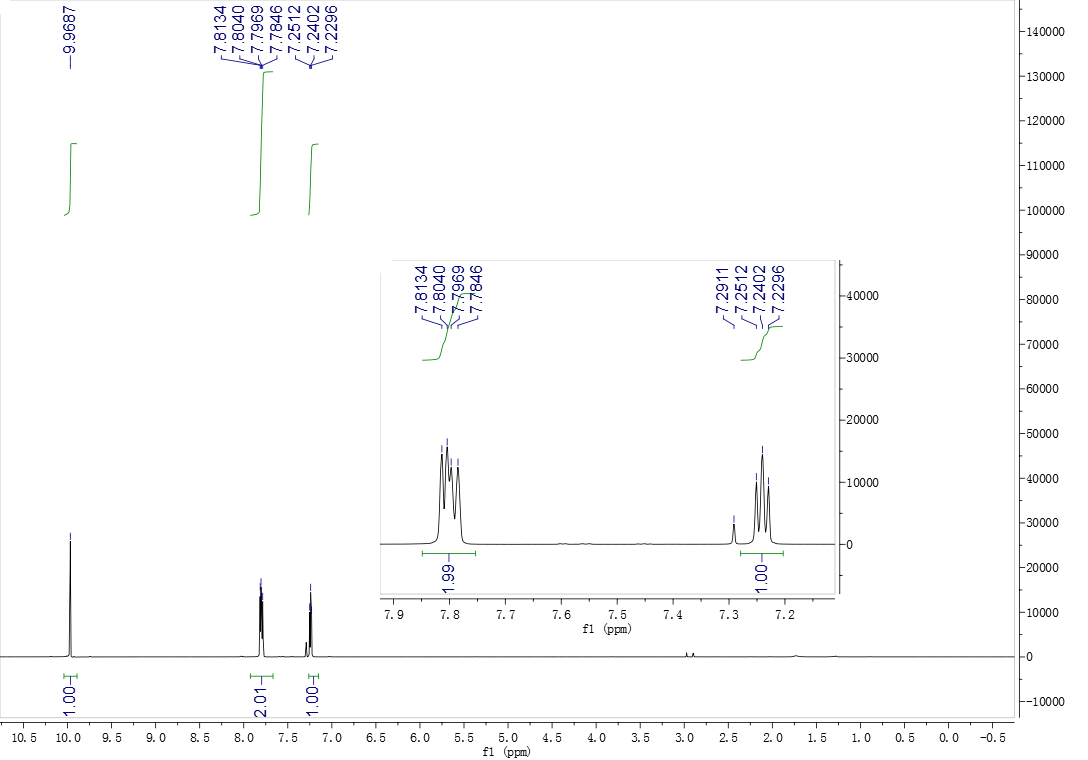


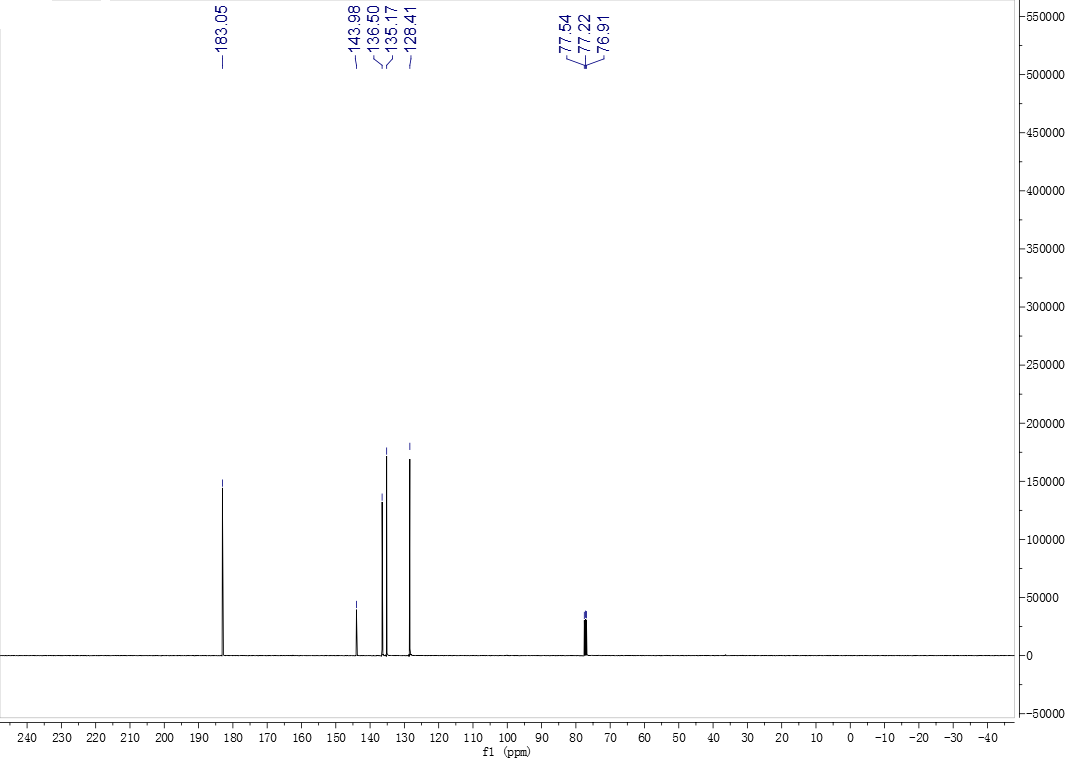


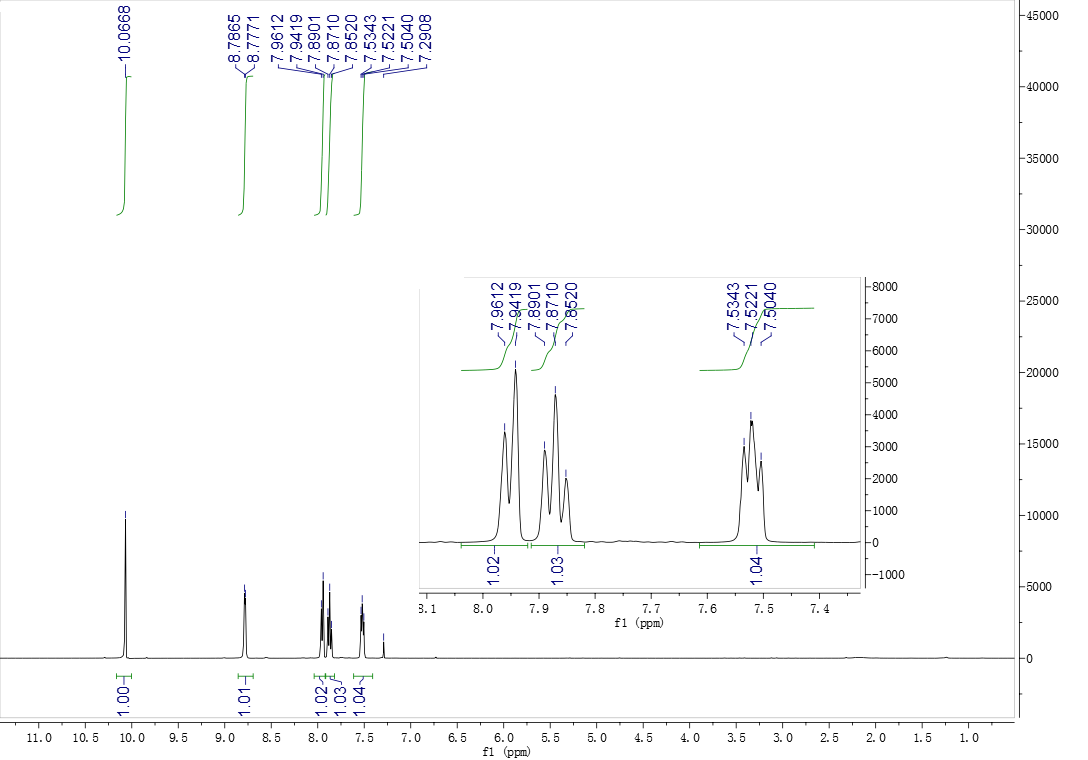


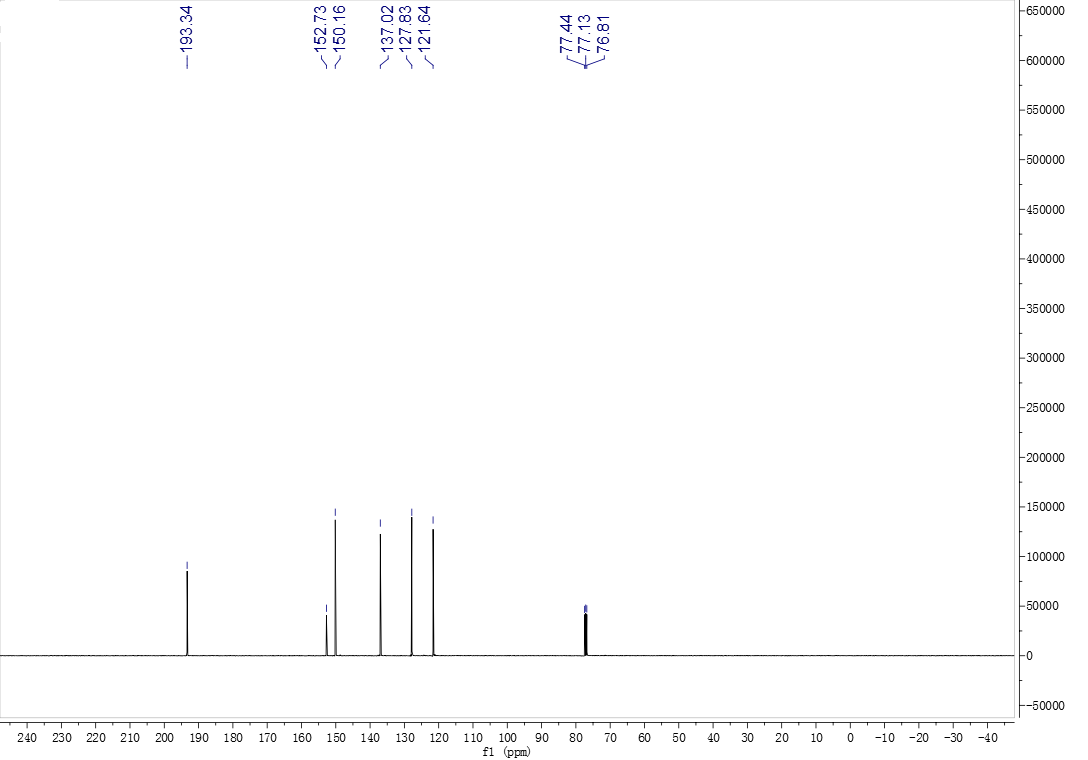


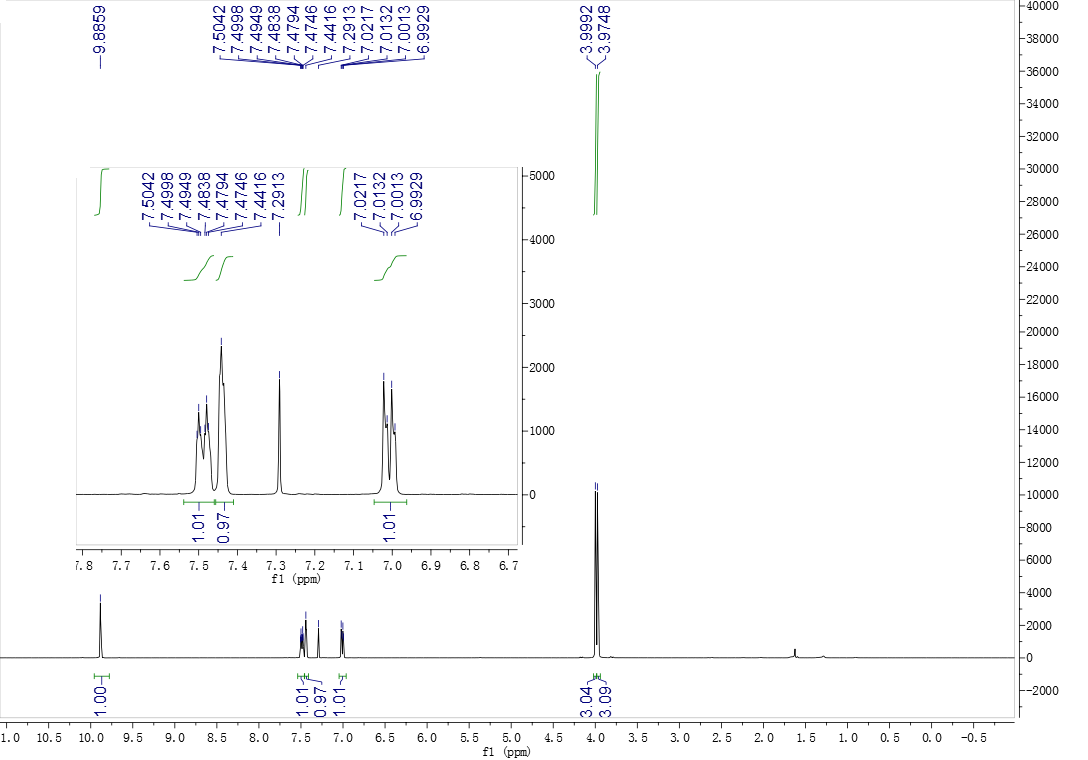


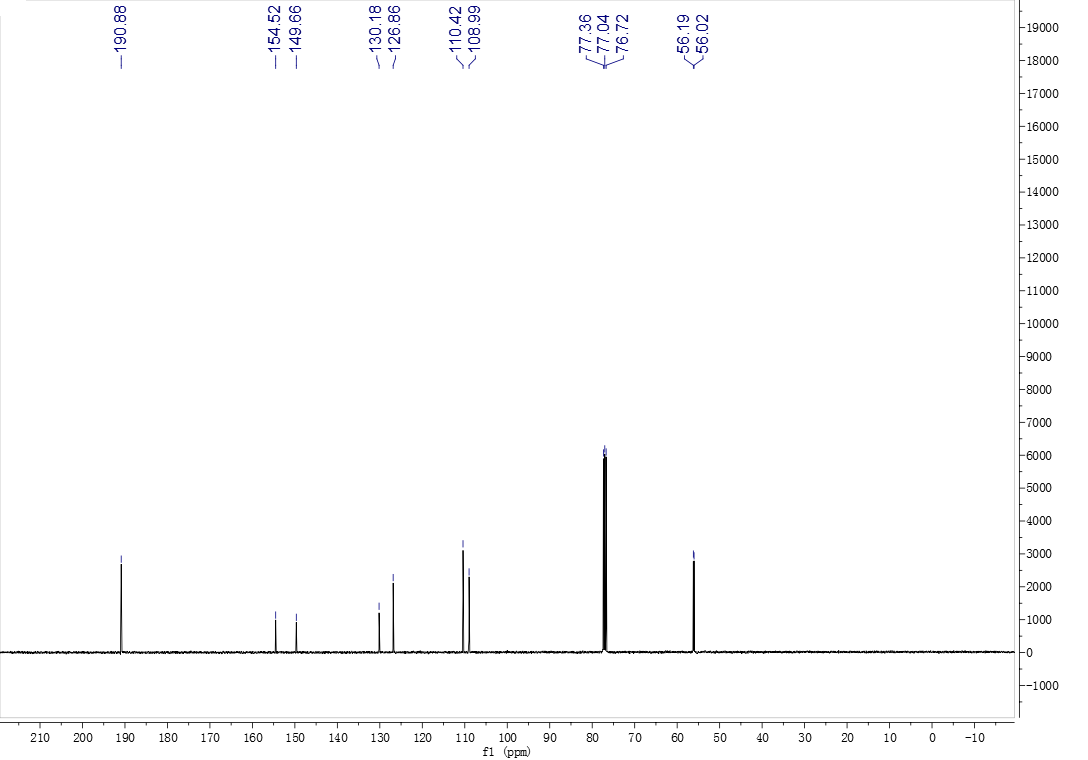


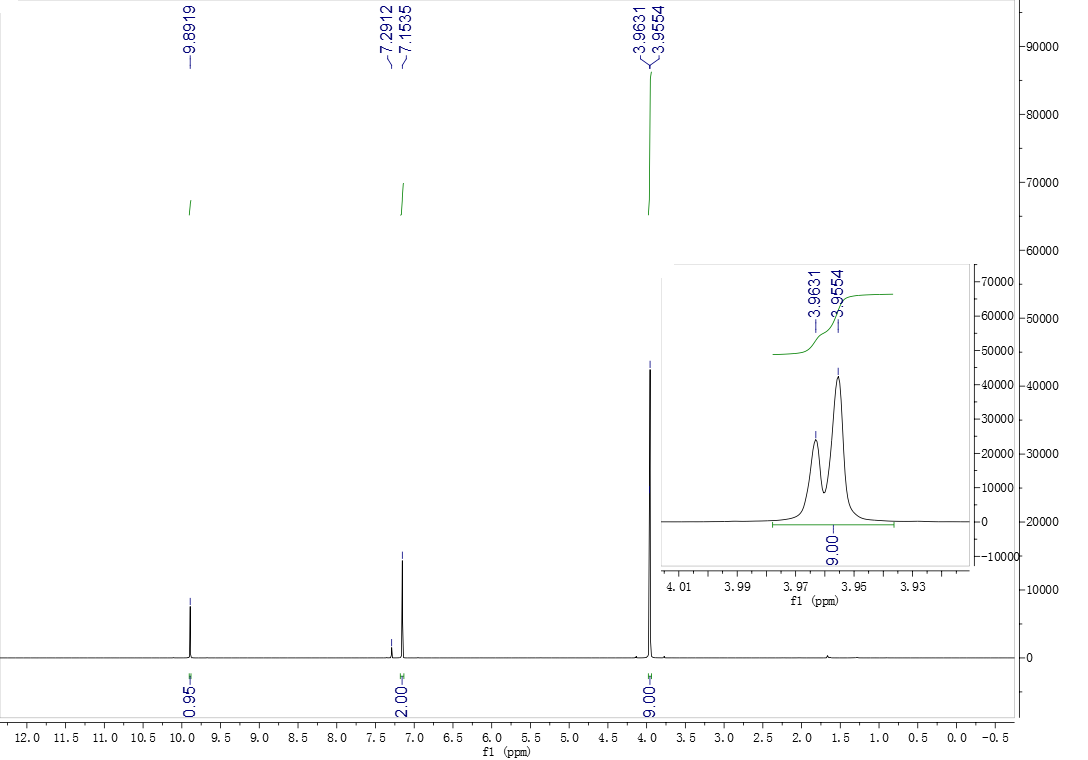


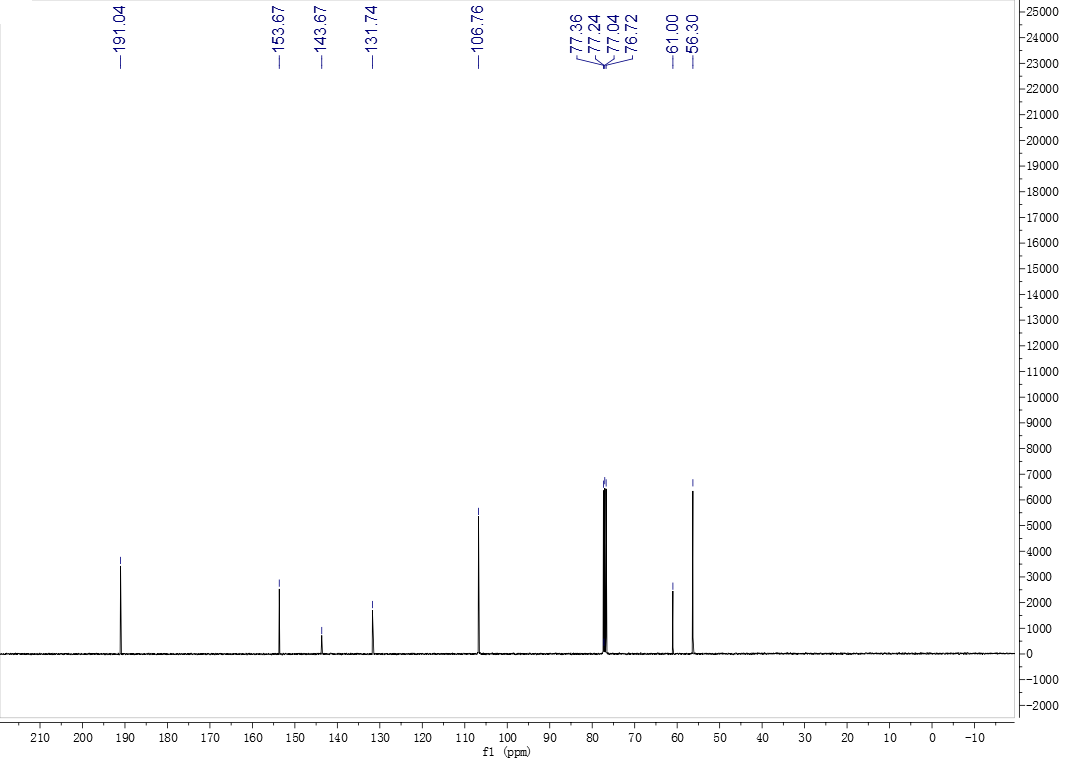


4. HRMS spectra

[SO_4_^2-^+H]^-^: calculated: 96.9596, found: 96.9600.

5. EPR spectra (Figure S1 and S2)


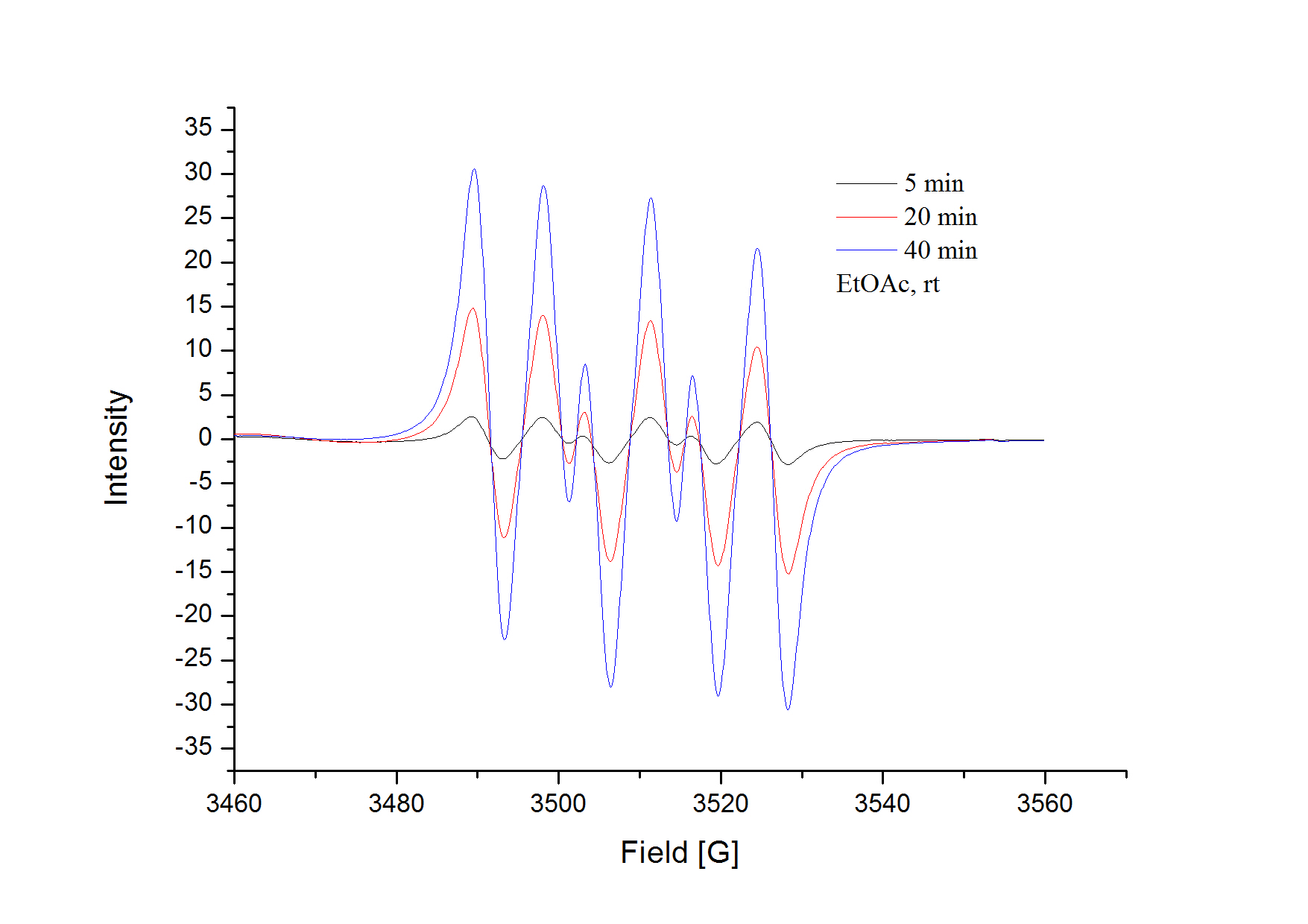


**Figure 1.** EPR spectra of alcohol **1a** (0.5 mmol), Na_2_S_2_O_4_ (174 mg, 1.0 mmol), DMPO (113.16 mg, 1.0 mmol) and *tert*-butyl hydroperoxide (257 mg, 2.0 mmol, 70% in water) in ethyl acetate at room temperature taken at t = 5 min, t = 20 min and t = 40 min.


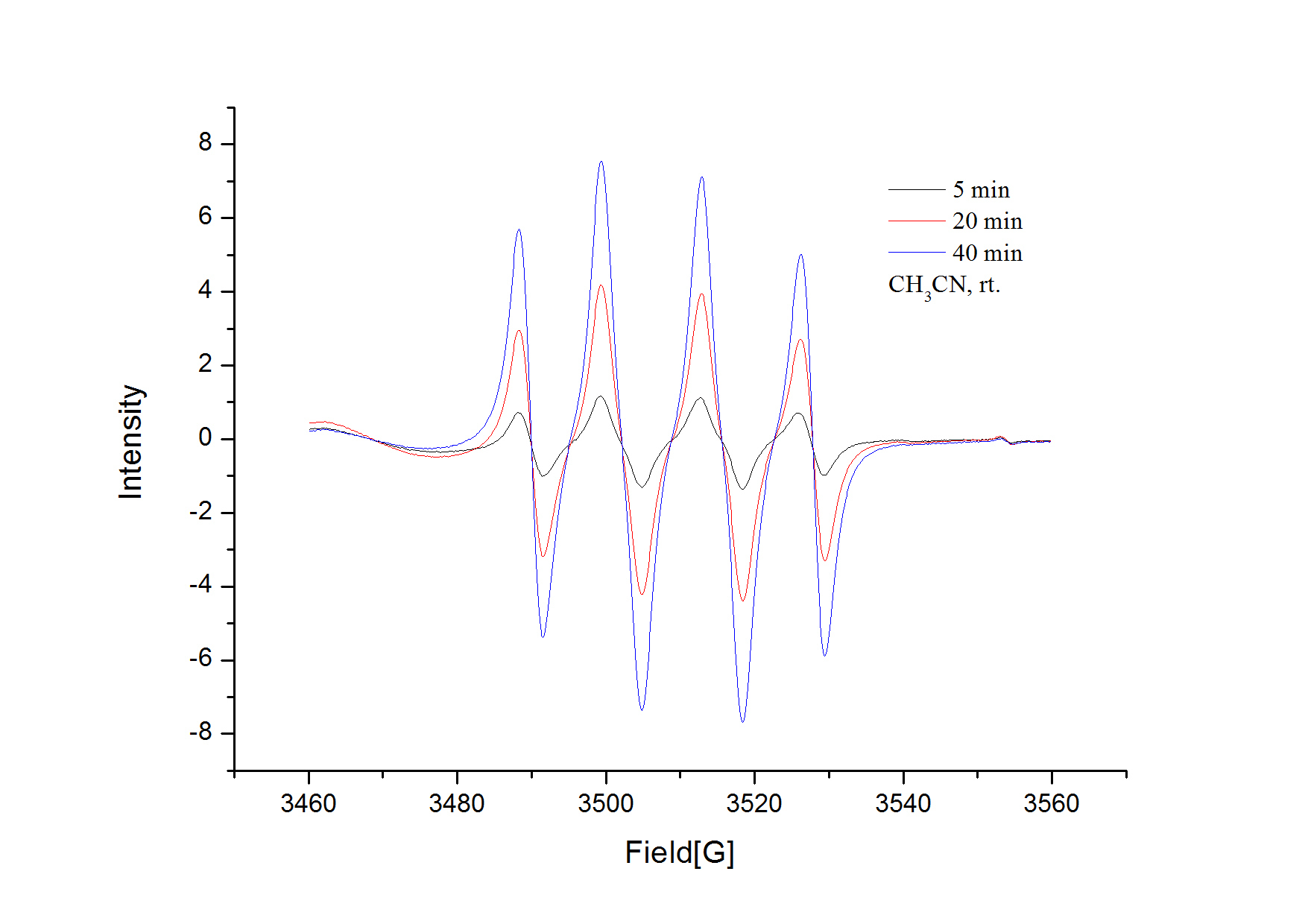


**Figure 2.** EPR spectra of alcohol **1a** (0.5 mmol), Na_2_S_2_O_4_ (174 mg, 1.0 mmol), DMPO (113.16 mg, 1.0 mmol) and *tert*-butyl hydroperoxide (257 mg, 2.0 mmol, 70% in water) in CH_3_CN at room temperature taken at t = 5 min, t = 20 min and t = 40 min.
